# Velocity Measurements in Channel Gas Flows in the Slip Regime by means of Molecular Tagging Velocimetry

**DOI:** 10.3390/mi11040374

**Published:** 2020-04-02

**Authors:** Dominique Fratantonio, Marcos Rojas-Cárdenas, Christine Barrot, Lucien Baldas, Stéphane Colin

**Affiliations:** 1 Los Alamos National Laboratories, Physics Division, Los Alamos, NM 87545, USA; 2 Institut Clément Ader (ICA), Université de Toulouse CNRS, INSA, ISAE-SUPAERO, Mines-Albi, UPS, 31400 Toulouse, France; marcos.rojas@insa-toulouse.fr (M.R.-C.); christine.barrot@insa-toulouse.fr (C.B.); lucien.baldas@insa-toulouse.fr (L.B.); stephane.colin@insa-toulouse.fr (S.C.)

**Keywords:** rarefied gas, slip velocity, channel flow, molecular tagging velocimetry

## Abstract

Direct measurements of the slip velocity in rarefied gas flows produced by local thermodynamic non-equilibrium at the wall represent crucial information for the validation of existing theoretical and numerical models. In this work, molecular tagging velocimetry (MTV) by direct phosphorescence is applied to argon and helium flows at low pressures in a 1-mm deep channel. MTV has provided accurate measurements of the molecular displacement of the gas at average pressures of the order of 1 kPa. To the best of our knowledge, this work reports the very first flow visualizations of a gas in a confined domain and in the slip flow regime, with Knudsen numbers up to 0.014. MTV is cross-validated with mass flowrate measurements by the constant volume technique. The two diagnostic methods are applied simultaneously, and the measurements in terms of average velocity at the test section are in good agreement. Moreover, preliminary results of the slip velocity at the wall are computed from the MTV data by means of a reconstruction method.

## 1. Introduction

In the last twenty years, microelectromechanical systems (MEMS) have received a lot of attention due to their appealing properties of low volume, low weight, low energy consumption, and system integrability. The development of new microfabrication techniques has made the design of novel MEMS possible, highly increasing the range of possible practical applications. There are many examples of MEMS that found a commercial application and that are present in many of our daily life utilities, such as microaccelerometers for smartphones, micronozzles [1] for space applications, microactuators [2] for aeronautical applications, micro heat exchangers [3], and Knudsen pumps [4], to name just a few.

Thus, from a scientific point of view, several new interesting physical problematics have arisen. Microsystems behave quite differently from their corresponding macro versions. As the device becomes smaller, the inertial forces decrease, and surface forces tend to gain importance with respect to volume forces. In gas microflows, the dynamics at the molecular level can lead to local non-equilibrium that affects the macroscopic thermodynamic properties of the gas flow. This microeffect is called rarefaction, and it is a consequence of the relatively low number of intermolecular collisions inside the control volume. The Knudsen number, $Kn=\lambda /L_{c}$, where *λ* is the molecular mean free path and $L_{c}$ is the characteristic size of the system, identifies the degree of gas rarefaction. Most of the microfluidic devices operate in slightly rarefied gas conditions, with *Kn* in the range ($10^{-3};10^{-1}$), which corresponds to the slip flow regime. This regime can be attained either in microfluidic devices with a low characteristic length $L_{c}$ or in larger systems at low pressure. In this rarefaction regime, the classical continuum representation of the flow is still acceptable for fluid particles that are far enough from the system’s solid boundaries, i.e., at a distance larger than the well-known Knudsen layer thickness, which is considered to be of the same order of magnitude of *λ*. For rarefied fluid systems that involve wall–gas interactions, the number of collisions between the gas molecules and the wall surfaces gains importance with respect to intermolecular collisions, thus producing a thermodynamic non-equilibrium state of the gas in the vicinity of the wall. At a macroscopic level, this produces a velocity slip and a temperature jump at the wall [5], i.e., non-negligible discontinuities of the kinematic and thermodynamic properties between surface and fluid. These rarefaction effects strongly influence the heat and mass transfers. Therefore, a correct modeling of the thermodynamic disequilibrium at the wall is crucial for an accurate prediction of the mass flow rates and heat fluxes within the microdevice. Experimental observations and analysis of the rarefaction effects are needed for the validation and the uncertainty quantification of the numerous theoretical and numerical analyses that already exist. However, most of the experimental analyses available in the literature on rarefied gas flows in channels are carried out by measuring global quantities, such as the mass flow rate, inlet and outlet pressures, and temperatures, to indirectly quantify the effects given by the velocity slip and temperature jump at the wall [6,7].

**Figure 1:**
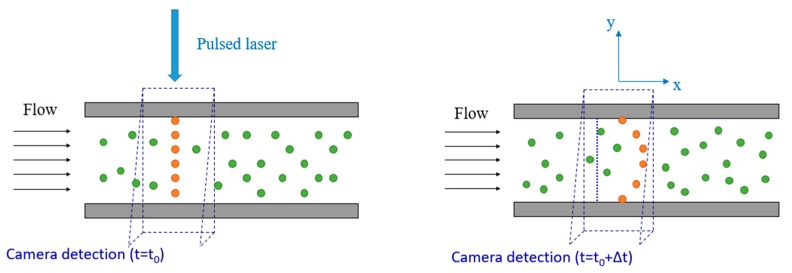
Basic principle of 1D-molecular tagging velocimetry (MTV) by direct phosphorescence, for a gas flowing in a plane channel from left to right.

To the best of our knowledge, there are no experimental data in the literature that provide local information on the velocity field in confined rarefied gas flows. In this context, we have developed an optical velocimetry technique with the final aim of applying it to rarefied gas flows inside a channel. Molecular tagging velocimetry (MTV) is an optochemical, low-intrusive technique capable of providing local measurements of the velocity field in gas flows. The technique is based on the tracking of the displacement of a suitable molecular tracer that exhibits photoluminescence properties. While various versions of this technique currently exist [8,9], this work demonstrates the implementation of 1D-MTV by direct phosphorescence, which is based on the long-lived phosphorescence emission of specific molecules, which can be activated by a single UV laser line excitation. In this work, the molecular tracer used is acetone $\left(CH_{3}COCH_{3}\right)$ vapor, which has a pressure vapor of 24 kPa at 20 °C and is recognized to have low toxicity if inhaled. A pulsed laser source tags a region of interest inside a gas flow and a photodetector captures the light emitted by the tracer at two successive times. The basic functioning principle of 1D-MTV in a plane channel is schematized in Figure 1. At the reference time $t_{0}$ a pulsed laser beam tags the initial reference position of the tracer molecules along a line perpendicular to the main flow direction. As the tracer moves by following the flow streamlines, the initial tagged line starts to deform accordingly to the velocity profile of the gas. The photodetector captures an image of the reference position at $t_{0}$ and an image of the deformed tagged line at a time $t_{0}+\Delta t$, where Δt is called the delay. The velocity profile can then be inferred from the molecular displacement profile $s_{x}\left(y\right)$ of the tagged line.

MTV is often considered as the molecular counterpart of particle image velocimetry (PIV). The substantial difference between the two velocimetry techniques relies on the nature of the tracer. MTV uses molecular seeding, which does not involve the complications specific to particle-based techniques, such as the relatively high response time of the particles, the difficulties in generating submicrometric sized particles, and the inaccuracies introduced by the Brownian motion noise. These issues make PIV inaccurate for applications in confined rarefied gas flows. For this reason, MTV is currently the most promising technique for flow visualization in microdevices and for accurate measurements of slip velocities at the wall. Nevertheless, while the MTV technique has already been successfully applied in various gas flows systems [10,11], the application of MTV to the case of confined and rarefied gas flows has been prevented up to now by various technological challenges. MTV measurements of fluid microflows require the adoption of short laser pulses at relatively high energy with a beam diameter smaller than the characteristic length of the system. Since diffraction phenomena limit the minimum laser beam waist that can be physically obtained by means of focusing optical lenses to about 30–150 µm, the channel height cannot be smaller than about 1 mm in order to be able to accurately resolve the strong velocity gradients. Hence, in order to reach Knudsen numbers in the slip flow regime, the average pressure needs to be lowered down to about 1 kPa. Unfortunately, low average pressures reduce the amount of tracer molecules present in the gas flow and increase the molecular diffusion of the tracer through the background gas. The combination of these two low-pressure effects makes the light signal disappear much faster than in high-pressure conditions, thus making MTV applications in rarefied gas flows very challenging.

In previous works we demonstrated that MTV could provide good results in a millimetric rectangular channel for a non-rarefied gas flow at atmospheric pressure and ambient temperature [12]. However, a combined effect of advection and molecular diffusion of the tracer in the background gas flow produces an important molecular displacement at the wall that is entirely independent of the actual slip velocity of the gas. Due to this phenomenon, known as Taylor dispersion, it is not possible to deduce correct values of the velocity profile by simply deriving the displacement profile with respect to time, not even in gas flows at atmospheric pressure. Subsequently, we have proposed a numerical method based on the advection–diffusion equation that was able to correctly reconstruct the velocity profile from the displacement of the tagged line. In a first step, the reliability of the method was proven by applying it to direct simulation Monte Carlo (DSMC) numerical experiments [13]. Later on, the method was proven to be valid also in the case of physical MTV experiments in confined non-rarefied flows. The experiments were performed in a millimetric channel at atmospheric and subatmospheric pressures down to a minimum average pressure of 42 kPa [14]. At this pressure level in a 1-mm deep channel, the flow was still in the continuum regime. In order to obtain velocity profile measurements in the slip flow regime, lower ranges of pressure are necessary. However, phosphorescence lifetime is highly reduced at such low pressures, and in order for MTV to work the tagged regions need to be interrogated on a time interval that is smaller than the lifetime of the tracer emission. In this respect, a very important step forward was realized by experimentally characterizing the photoluminescence properties of the tracer molecules in terms of signal intensity and lifetime at low pressures. The experiments performed on acetone and diacetyl vapors demonstrated that MTV measurements in confined rarefied gas flows should be possible [15].

Thus, by using all the knowledge acquired up to now on this subject, we present in this work optical experimental results of molecular displacement in the slip flow regime in a confined environment. To the best of our knowledge, the results here presented are the very first flow visualizations of gas displacement in a confined domain and in rarefied conditions. Moreover, they are the first visual proof of velocity slip at the wall in rarefied gas flows. MTV data are reported for argon and helium flows in a 1-mm deep channel with rectangular cross-section and at pressures between 30 and 0.9 kPa, thus reaching Knudsen number values up to Kn = 0.015. In this work, the Knudsen number Kn is defined with respect to the channel height H.

This manuscript is organized as follows. Section 2 describes in detail the experimental setup used for carrying out MTV measurements in low-pressure gas flows through a channel with a rectangular cross-section. In Section 3, the experimental methodology used for acquiring MTV images in slow-varying thermodynamic gas flow conditions is thoroughly explained and the advantages and disadvantages of this approach are critically discussed. Section 4 describes the sequence of used post-processing operations, from the recorded MTV images to the extraction of the displacement profile data to be used in the reconstruction method developed in [13]. In Section 5, the experimental results of this work are reported. Firstly, MTV data of argon and helium flows in non-rarefied and rarefied conditions are presented. Secondly, MTV data are compared with data obtained by the constant volume (CV) technique in terms of average velocity. Finally, the conclusions and future developments of this work are discussed in Section 6.

## 2. Experimental Setup 

**Figure 2:**
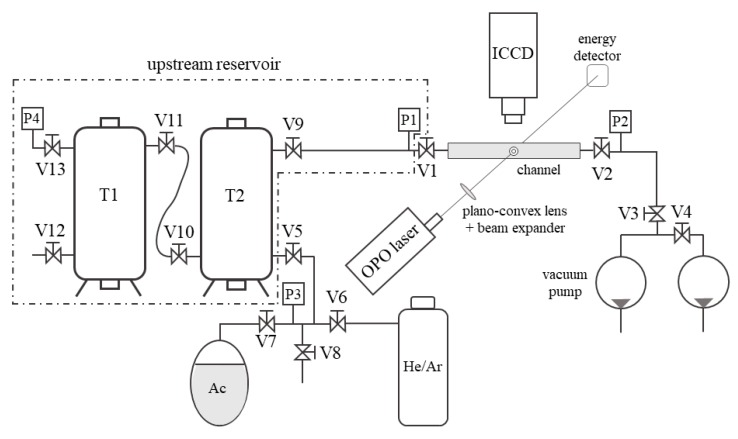
Gas system for application of MTV to the gas–vapor mixture flows at low pressures. T1 and T2 are two tanks; P are pressure sensors; V1 is the inlet channel valve; and V2 is the outlet channel valve.

The gas system designed and built in this work for applying MTV to gas flows at low pressures is illustrated in Figure 2. The basic working principle of this open loop gas system relies on (i) preparing the gas-tracer mixture in a big reservoir at the desired pressure $p_{1}$ and tracer concentration χ and (ii) forcing the gas mixture to pass through the channel by pumping it into the atmosphere. The upstream reservoir was composed of two tanks of about 90 L each. A pipe with a large diameter of about 1.5 cm and a length of 1.3 m connected the two tanks. At the outlet of the channel, two GEFI^®^ rotary vacuum pumps (Pfeiffer Vacuum, Aßlar, Germany) of the same model were used in parallel to achieve the highest mass flow rate through the channel. The pumping at the channel outlet was required for exhausting the gas from the tanks into the atmosphere since the experimental flow conditions were at pressures lower than the atmospheric one. The gas system was equipped with Inficon Sky^®^ CDG025D capacitance diaphragm gauges (Inficon, Bad Ragaz, Switzerland) for measuring the gas pressure at different locations. Pressure sensors P1 and P2 were used for monitoring the static pressure at the inlet and the outlet of the channel, respectively. Pressure sensor P3 on the mixing line was necessary for monitoring the complete outgassing of air in the acetone bottle. Pressure sensors P4 and P1 could be both used for measuring the pressure in the tanks. P4 could also be used during the experiments in order to verify that the pressure inside the tanks is at any time equal to the one at the entrance of the channel. For all the experiments carried out during this work, no difference was recorded between the measured $p_{1}$ and $p_{4}$ values. 

The choice of using an open loop gas circuit instead of an apparently simpler closed loop system is the result of several practical issues that a closed loop configuration has for the application of MTV. Firstly, it is difficult to find commercial pumps or fans that are simultaneously oil-free, leakage-free, and chemically resistant to acetone vapor, and, secondly, the tested channel itself is not perfectly free from air leakages. Since oxygen molecules are efficient quenchers of acetone phosphorescence [16,17], a fresh gas–acetone mixture needs to be introduced in the test section at each MTV acquisition, thus making the closed loop configuration an unsuitable solution. The main disadvantage in using an open loop gas system is that the thermodynamic flow conditions in the tested channel vary in time. The inlet–outlet pressure difference and the average pressure at the test section decreased in time as the upstream reservoir pressure decreased. This consideration justifies the use of a couple of big tanks for the upstream reservoir to minimize the variations in time of the flow conditions in the test section. The stability of the experimental conditions in relation to the MTV acquisitions was discussed in detail in Section 3. Nevertheless, a varying upstream gas pressure turned out to be an advantage as it allowed the simultaneous application of the CV technique for monitoring the mass flow rate through the channel. This simultaneous measurement technique provided further experimental data that was used for MTV data validation.

The channel was manufactured with (i) an optical access for the UV laser excitation of the tracer seeded in the gas flow; (ii) an optical access for capturing the phosphorescence emission of the tracer by means of an intensified CCD; (iii) materials that were chemically resistant to acetone vapor; and (iv) good sealing properties for preventing leakages at low pressures. Two PEEK^®^ plates (Victrex plc, Lancashire, UK) composed the main body of the channel. The channel was grooved inside the first plate, while the second one served as a cover. A planar joint of Kalrez^®^ (DuPont de Nemours, Inc., Wilmington, DE, USA) was positioned in between these two plates in order to prevent leakages.

**Figure 3:**
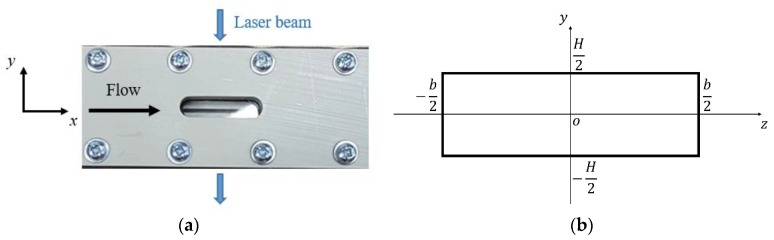
In (**a**), a global view of the test section along the channel from the point of the CCD camera. The blue arrows indicate the optical access for the laser beam and the black arrow shows the gas flow direction. In (**b**), a sketch of the channel cross-section along with the adopted Cartesian coordinate system.

**Figure 4:**
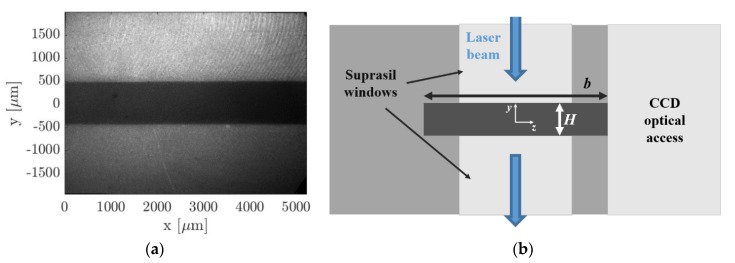
In (**a**), an image of the channel gap in the region of interest for MTV, recorded with the CCD. In (**b**), a sketch of the channel cross-section at the MTV test section. The upper and lower bright regions correspond to the Suprasil^®^ windows for the laser beam access. The bright region on the right represents the optical access for the CCD camera. The width of the cross-section and the distance between the Suprasil^®^ windows are indicated on the image as b and H, respectively.

Let us define a Cartesian coordinate system in which the cross-section of the channel lies on the y-z plane, and the *x*-coordinate represents the position along the channel length L. The inlet and the outlet of the channel corresponded, respectively, to x=0 and x=L, while the walls were located at y=±H/2 and z=±b/2, where H and b are the height and the width of the channel, respectively. The laser beam excitation was centered at x=L/2 and z=0 (Figure 3b). As shown in Figure 3a, an optical access in borosilicate, transparent to light in the visible spectrum, allowed the acquisition of the phosphorescence signal in the x-y plane. Figure 4a reports an image of the channel recorded by the CCD through the borosilicate glass. Two other windows were used for the laser beam access. They were fabricated in a special grade of Suprasil^®^ (Heraeus, Hanau, Germany), which is a high purity synthetic fused silica material highly transparent to UV light. The first window allowed the laser beam access to the channel section by one side and the second window allowed its exit by the other side. This double-window system prevented laser reflections in the interrogation region and allowed a continuous monitoring of the laser energy at the laser exit by means of an energy detector. Figure 4b illustrates a sketch of the structural composition of the channel cross-section in the y-z plane and at x=L/2. As it can be observed, the value of the channel height H at the position of the laser beam excitation directly depended on the actual distance between the two Suprasil^®^ windows.

Accurate measurements of the channel height H at the MTV test section are required for accurately extracting the local velocity profile from the MTV displacement data by means of the reconstruction method. For the computation of the average velocity from the mass flow rate measurements provided by the CV technique (described in Section 5), reliable measurements of both width b and height H at the MTV test section are necessary. With the help of tomographic imaging, the position of the Suprasil^®^ windows has been carefully adjusted to be in line with the walls of the channel grooved into the PEEK^®^ plate. Tomographic images also showed proper conditions of the PEEK^®^ and the windows surfaces. However, because of the low average pressure in the gas system used during MTV acquisitions, the windows position can vary slightly as a function of the pressure difference between the inside and the outside of the channel. Tomographic measurements could be made only with the channel at atmospheric pressure. The uncontrolled micrometric movements of the windows are then sources of uncertainty for the measured value of H. For this reason, an alternative solution has been considered for having the best estimation of the wall distance H. This approach was presented in detail in Section 4 and consisted of an image processing procedure that detects the real positions of the wall directly on MTV images. By this methodology, the channel height was measured to be H = 1003 µm, which was consistent with the value measured from tomographic images. The width b was measured to be 5000.8 µm with a standard deviation of 7.4 µm by tomographic measurements, and the length L of the channel was 20 cm.

For the molecular tagging system, an OPOlette HE355LD laser was used for all the experiments, since it allowed us to provide the proper wavelength excitation for acetone vapor, which was set to 310 nm. The laser pulses were shot at a rate of 20 Hz and last 7 ns each. The diameter of the beam was focused into the interrogation area down to about 150 μm. The beam diameter was here defined as the full width at half-maximum, i.e., the width at which the laser energy was half of the central peak value. After UV excitation, acetone vapor emitted a certain amount of light that could be described as the sum of two components: (i) a strong light emission that lasts for only some nanoseconds, which is commonly defined as fluorescence, and (ii) a less intense light emission that can last up to a millisecond, which is known as phosphorescence. The definitions of fluorescence and phosphorescence emissions are not based on the intensity and the duration of the emission but on the type of intramolecular electronic transition that makes an excited molecule come back to its ground electronic state [18]. An extensive analysis of the dependency of acetone phosphorescence intensity and lifetime on excitation wavelength and pressure is discussed in Fratantonio et al.’s research [15]. The laser beam diameter has been estimated from images of the fluorescence intensity distribution.

A 12-bit Imager Intense (LaVision^®^) progressive scan CCD coupled with a 25-mm intensified relay optics (IRO) was employed for the image recording. The IRO was made of a S20 type photocathode and a P46 phosphor plate. The CCD was composed of 1376 photodiodes × 1040 photodiodes. Due to the very low intensity of the phosphorescence emission, a binning process is required for increasing the sensitivity to light of the CCD at the expense of reducing the spatial resolution. A 4 × 4 binning was used for all the experimental data presented in this work; the resolution of the CCD was thus reduced with a total of 344 pixels × 260 pixels, each pixel corresponding to 16 photodiodes. A 105 mm f: 2.8 and an inverted 28 mm f: 2.8 Micro Nikkor lenses (Nikon Inc., Tokyo, Japan) were used for collecting the light emitted by the acetone molecules. The external optical system and the internal optical collector of the IRO and CCD provided an overall magnification of about 1.7. As the CCD covered an actual area of 8.87 mm × 6.71 mm, the field of view was 5.29 mm × 4 mm. Consequently, each pixel corresponded to an area of 3.8 μm × 3.8 μm without binning and to 15.2 μm × 15.2 μm with a 4 × 4 binning. A programmable timing unit (PTU) was used for synchronizing the laser trigger, the camera shutter, and the IRO trigger. The main parameters that can be controlled for each record are the IRO gate $\Delta t_{gate}$, i.e., the time interval of light integration, the IRO amplification gain *G*, the delay Δt between the IRO trigger and the laser excitation, and the exposition time $t_{CCD}$ of the CCD detector.

The low phosphorescence emission rate and the low amount of tracer molecules seeded in a low-pressure gas flow made the amount of light emitted after one single laser excitation very low. In addition, the laser energy level needs to be limited to avoid damaging the Suprasil^®^ windows. If the amount of light collected from one laser excitation is too low to stand out from the CCD background noise, averaging more images does not help in increasing the signal intensity. For this reason, the on-chip integration was used, which allowed us to collect in one single image the light generated by more than one laser excitation. The final image is the result of averaging $N_{i}$ images, each one integrating $N_{l}$ laser pulses. A higher number $N_{i}$ of averaged images can increase the quality of the resulting image by reducing the data fluctuations, and thus increases the signal-to-noise ratio (SNR). More information on the signal acquisition can be found in Fratantonio et al.’s research [15]. The uncertainty on the velocity measurement depends on the accuracy of the measured molecular displacement and the time separation between two images. 

## 3. Experimental Methodology

In order to be able to measure a sufficiently deformed tagged line by means of MTV, stationary and relatively high flow rates are needed. The desired velocity magnitudes through the test section were obtained by imposing large pressure differences between the inlet and the outlet of the channel. Nevertheless, the MTV experiments were performed in time-dependent experimental conditions, since the imposed pressure difference was not constant with time. However, as it will be demonstrated in this section, the flow conditions were stable enough during the time needed to perform one MTV acquisition.

**Figure 5:**
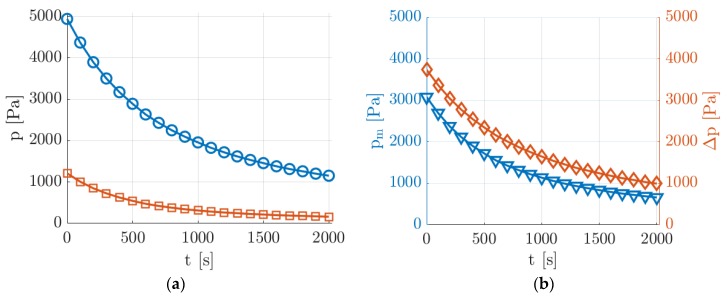
Evolution in time of the experimental pressure conditions generated at low pressures acquired by pressure sensors P1 and P2: (**a**) upstream $p_{1}$ (O) and downstream $p_{2}$ (**☐**) pressure measurements and (**b**) mean pressure $p_{m}=\frac{p_{1}+p_{2}}{2}$ (**▽**) and pressure difference $\Delta p=p_{1}-p_{2}$ (**◇**).

With reference to the sketch of Figure 2, the preparation of an experimental run conducted at low pressure was done by filling the upstream reservoir (tanks T1 plus T2) with a gas mixture at a pressure $p_{1}$ of about 5 kPa and by vacuuming to a minimal pressure the volume downstream from valve V1, which was initially closed. When opening valve V1 at the channel inlet, the gas mixture started flowing through the channel and the downstream pressure $p_{2}$ quickly increased to about 1.2 kPa, as a consequence of the limited maximum mass flow rate that the two pumps could sustain at the working pressure $p_{2}$. Figure 5 shows the typical evolution in time of the pressure conditions applied to the tested channel during one experimental run at low pressure.

The average pressure $p_{m}$ in the channel varied between 3000 and 700 Pa with a corresponding pressure difference Δp that decreased from 4000 to 1000 Pa. For a helium–acetone flow with acetone molar concentration of χ = 20%, this pressure range corresponded to a value of Kn at the test section varying from 0.004 to 0.018. The decrease of $p_{m}$ and Δp slowed down as the average pressure in the upstream reservoir decreased since the mass flow rate through the channel decreased as well. The slowly decreasing downstream pressure was beneficial for the stability of the velocity profile to be measured. The magnitude of the velocity profile was strongly dependent on the pressure gradient, and the fact that the upstream and downstream pressures decreased simultaneously made the pressure gradient vary slower. Moreover, as the pressure in the system decreased, the pressure in the upstream tanks varied slower as well because of the decreasing mass flow rate through the tested channel. At the lowest average pressure, the flow speed was thus much more stable, which is a suitable experimental condition for the application of MTV. Even though the experimental run could continue towards even lower pressures than those reported in Figure 5, this work presents MTV results only down to a minimum pressure of about 900 Pa.

**Figure 6:**
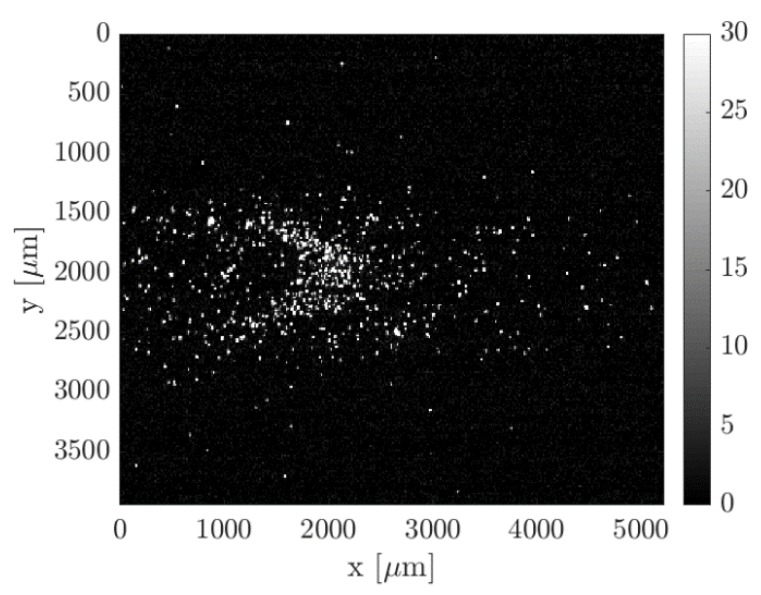
Example of a raw image acquisition of the acetone emission in argon flow at $p_{m}$ = 30 kPa, Δp = 550 Pa, and an acetone molar concentration χ = 20%. The image results from the integration of $N_{l}$ = 100 laser excitations, with delay Δt = 50 µs, gain G= 100%, IRO gate $\Delta t_{gate}=$ 100 ns, and 4 × 4 binning.

The application of MTV for the investigation of the velocity field requires the gas flow to be steady or at least slowly varying in time. The evolution in time of the investigated velocity profile during one experimental run could be predicted and its rate of variation could be compared with the time requirements of the acquisition system. The recording of one image with $N_{l}$ = 100 and with a laser repetition rate of 20 Hz took 5 s. From the pressure measurements, it was possible to calculate that the velocity profile varied by less than 0.5% during the recording of one single image at any time along the experimental run. This fact confirms that the experimental setup was able to generate experimental flow conditions that were in the slip regime and that were stable enough for MTV acquisitions. Nevertheless, the SNR of one single recorded image was too low to provide an accurate measurement of the tagged line displacement. Figure 6 shows an example of one single image collecting 100 laser pulses with an intensifier IRO gate $\Delta t_{gate}$ = 100 ns per laser shot.

**Figure 7:**
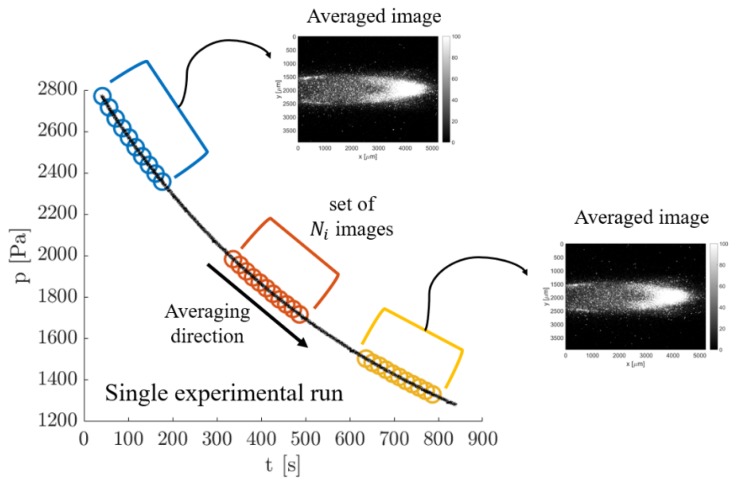
Qualitative illustration of the strategy used for producing an averaged image from a group of $N_{i}$ images. The average is made “in cascade” on $N_{i}$ images recorded during the same experimental run.

Exploitable results for carrying out the velocity measurement require averaging several images representing the same flow conditions. A possible strategy consists of averaging consecutive images recorded along one experimental run by taking advantage of the slow variation in time of the investigated flow conditions. Since one complete experimental run lasted about 30 min and the laser repetition rate had a frequency of 20 Hz, a group of about 360 images could be collected for $N_{l}$ = 100 laser shots per image. While for a time frame duration of one image the thermodynamic conditions were substantially the same, different images recorded along one experimental run were related to different average pressures and flow rate conditions. Figure 7 illustrates the averaging procedure used.

By recording images with the same delay Δt during one complete experimental run, the average could be applied to any group of $N_{i}$ images that belongs to a time window centered around the experimental condition of interest. By moving the averaging window along the experimental run, a great number of averaged images that represent a multitude of thermodynamic conditions could be obtained from one single experiment. Thus, this averaging strategy could be considered to be not very time-consuming since a high number of averaged images at a specific delay Δt and for different average pressures and pressure differences could be obtained in a 30-min experimental run.

In order to obtain measurements at different delays, several experimental runs with the same initial boundary conditions need to be launched. This has to be done in order to capture the displacement profile of the same flow conditions but at different times after the laser excitation. For vacuum conditions at the outlet, the only parameter that needs to be regulated at the beginning of the experiment is the upstream pressure $p_{1}$. Therefore, the same initial boundary conditions could be easily reset for different experimental runs. The repeatability of the experimental conditions is important for both the mass flow rate measurements provided by the CV technique (see Section 5) and the velocity measurements provided by MTV. For the former technique, the possibility of reproducing the same experimental conditions allowed us to repeat multiple times the mass flow rate measurements, thus helping in characterizing its statistical uncertainty. For MTV, the fact that the same experimental flow can be reproduced with the highest accuracy is, instead, essential for comparing velocity measurements that come from displacement profiles recorded at different delays after the laser excitation. By repeating several times experimental runs with the same initial conditions of pressure and gas composition in the upstream tanks, it could be ascertained that the gas circuit used in this work was able to reproduce the same flow conditions along the whole duration of the experimental run and that no appreciable differences were present from one experiment to another.

**Figure 8:**
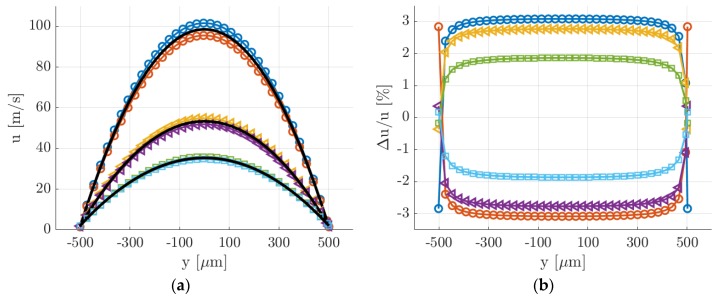
(**a**) Analytical velocity profiles u(y,t) at t = 100 s (O), 1000 s (◁), and 2000 s (**☐**) during the experimental run. The evolution of u(y,t) during the recording of $N_{i}$ = 20 consecutive images is illustrated. At each time t, the figure reports: the velocity profile captured by the first image of the group; the velocity profile captured by the last image of the group; the average velocity profile (solid black lines) that the image resulting from the average of the $N_{i}$ images would represent. (**b**) Relative variation of the velocity profile with respect to the average velocity profile at t = 100 s (O), 1000 s (◁), and 2000 s (**☐**).

The drawback of this averaging strategy is, however, the fact that the group of $N_{i}$ images used for producing one averaged image corresponded to slightly different thermodynamic conditions. The group of images could not be, therefore, arbitrary large. The size of the averaging window needs to be small enough, so that the experimental conditions of the investigated flow vary only in an acceptable range. In this perspective, it is of fundamental importance to assess how much the experimental conditions vary from one image to the other. The MTV results presented in Section 6 were performed with $N_{i}$ = 20, therefore the following analysis was presented only for this number of averaged images. By considering that the time between two consecutive image acquisitions was 5 s, the recording of $N_{i}$ = 20 images took 150 s. In Figure 8, the velocity profiles together with their variations in respect to time were calculated at t = 100, 1000, and 2000 s from the beginning of a representative experimental run (the same as in Figure 5). The velocity profiles were computed from the slip flow analytical solution proposed by Ebert and Sparrow [19] by assuming a tangential momentum accommodation coefficient (TMAC) equal to 1. 

At t = 0, that is for the highest pressure differences imposed and average pressures, the velocity variations with time were the highest, with a maximum value of about 3%. At t = 2000 s, that is for the lowest pressures, the velocity variations with time were the lowest, with a maximum variation of less than 2% at the velocity centerline. Consequently, the time stability of the velocity profile was lower at higher pressures. As the average pressure in the system decreased and the slip regime was approached, the velocity profile varied slower. Moreover, while the centerline velocity substantially decreased during one complete experimental run, the slip velocity at the wall was much more stable in time and varied from 1.6 m/s at t = 0 to 2.2 m/s at t = 2000 s. This peculiarity of the experimental flow conditions is of great help for the validation of MTV velocity reconstructions. As the Knudsen number at the test section increased, the ratio of the slip velocity to the average velocity was expected to raise from 2.5% to 10%.

## 4. MTV Image Post-processing

**Figure 9:**
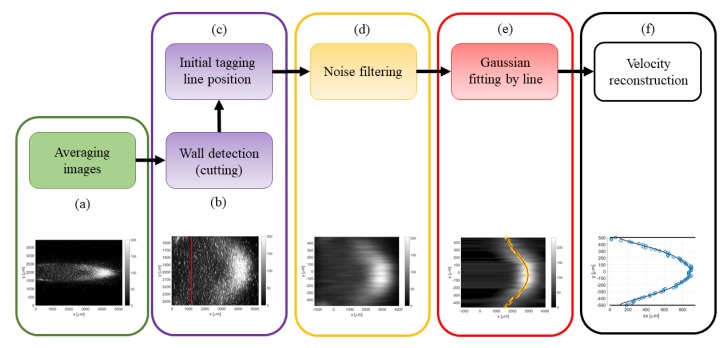
Flow chart of the image post-processing: (**a**) image averaging; (**b**) detecting wall position and image cutting; (**c**) detecting tagged line position; (**d**) filtering background noise; (**e**) Gaussian fitting per each horizontal line of pixels; and (**f**) velocity reconstruction from the displacement data $s_{x}{}_{j}$.

The main objective of the post processing procedure was to identify the position and the shape of the deformed tagged line. Several steps are necessary for accurately extracting the displacement profile from a noisy image. The flow chart in Figure 9 shows the sequence of post-processing operations, which are image averaging, wall detection, detection of the tagged line initial position, filtering of the background noise, Gaussian fitting per line, and extraction of the displacement data $s_{x}{}_{j}$. In step (a), an example of image resulting from averaging $N_{i}$ images is shown. Steps (b) and (c) are grouped, as they are operations that simply modify the size of the image or the Cartesian coordinate system origin. In step (d), MTV images are passed through a proper noise filter to make the fitting procedure of step (e) more robust. Finally, step (f) consists in the actual measurement of the velocity profile by means of the reconstruction method [13]. All the operations described below were implemented by means of Matlab^®^ software (The MathWorks Inc., Natick, MA, USA) and were systematically applied to all the recorded images. The way step (a) is performed was thoroughly described in the previous section. The next steps of the procedure are discussed below in detail.

**Figure 10:**
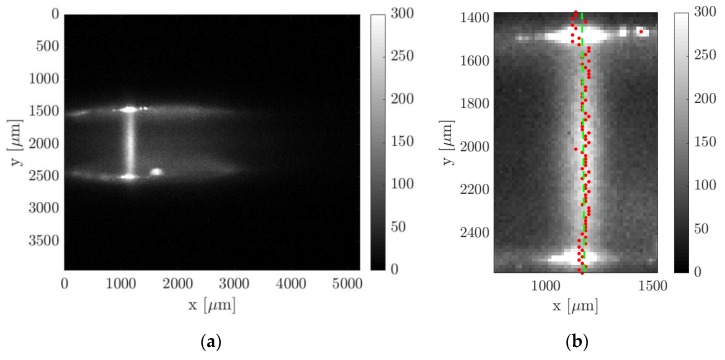
Post-processing procedure for detecting the initial position of the tagged line: (**a**) raw image of acetone early emission and (**b**) fitting of the maximum intensity along the *y*-coordinate detected in the region of interest.

**Figure 11:**
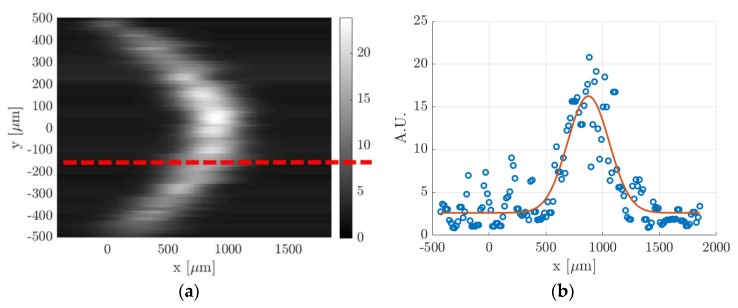
(**a**) Image resulting from the application of the Gaussian fitting per line to a raw MTV image and (**b**) example of data distribution along a horizontal line of pixels, indicated by the red dashed line in (**a**), and the corresponding Gaussian fitting (A.U. stands for arbitrary unit).

(b) Wall detection: the strategy employed in this work for the identification of the channel walls was based on the investigation of the light emission provided by the top and bottom Suprasil^®^ windows. The fluorescence of the silica is short-lived but very strong in the first few nanoseconds after the laser excitation. By recording the light emission of the silica with no tracer vapor inside the channel, the spots of maximum intensity on the windows correspond to the locations of entrance and exit of the laser beam, and, therefore, they represent the position on the image of the intersection between the tagged line and the channel walls. The procedure described above has a limited precision in localizing the channel walls. Firstly, the lowest possible uncertainty is determined by the pixel size. Secondly, the maximum intensity emission on the Suprasil^®^ windows represents only an indication of where the wall is located. The identified wall lines may correspond to the solid surface that is just before or just after the plane where the tagged line is developing. Nevertheless, this strategy for detecting the wall position on the image is currently the most accurate one available.

(c) Initial tagged line position: the measurement of the displacement profile along the channel height requires first the identification of the initial position of the tagged molecules. In order to minimize possible displacements of the vertical tagged line from its original position, the flow rate needs to be low enough with respect to the chosen delay of acquisition. The interest in slightly delaying the time of acquisition with respect to the laser excitation derives from the idea of getting rid of the intense short-lived emission of Suprasil^®^. Based on the gas flow speed, the delay of acquisition needs to be chosen small enough to maintain the displacement of the tagged line at least smaller than 1 pixel. For a gas flow with an average velocity of 30 m/s, the delay cannot be higher than 100 ns or 500 ns, respectively whether no binning or a 4 × 4 binning is used. The post-processing procedure for the computation of the tagged line position is quite trivial. Figure 10 illustrates an example of early emission from the tagged molecules. Firstly, a region of interest is defined around the tagged line and, secondly, the position of the maximum light intensity is localized along the tagged line. A linear fitting of the light peaks provides the mean abscissa $\mu_{x}$ of the tagged line and a possible inclination of the laser beam with respect to the wall surface. Since the SNR is relatively high, it is preferred to avoid the use of binning for obtaining the highest precision in the evaluation of $\mu_{x}$. By assuming that the laser beam position does not move with respect to the CCD, the abscissa $\mu_{x}$ is considered as the starting position of the tagged molecules for the images representing the deformed tagged line. The *x*-coordinate system of these images is translated so that the origin x = 0 corresponds to the initial position of the tagged line.

(d) Noise filtering: the identification of the deformed tagged line is based on the application of appropriate fitting functions on the recorded image. The fitting procedure is a tool for extracting the most important information out of the background noise. Nevertheless, the SNR of the image is sometimes so low that the background noise prevents the convergence of the fitting optimization. In these cases, it is necessary to first pretreat the image with a noise filter for improving its SNR. A first strategy for reducing the background noise is based on the application of a 2D filter, which is a filter function applied on the whole image. The type of 2D filter employed in this work is the median filter, which is very efficient in removing the speckle noise. A second possibility consists of applying a 1D noise filter on each horizontal line of pixels. A family of digital filters that can be employed for this purpose is the finite impulse response (FIR) filters family. Both the 2D median filter and the 1D FIR filters have been used to improve the SNR of the image. However, the median filter has shown to be more efficient in improving the convergence of the fitting procedure while maintaining the sharpness of the tagged line.

(e) Gaussian fitting per line: in order to track the molecular displacement at different times from the initial position, an appropriate criterion for identifying the tagged line profile is required. Previous analyses have shown that the best fitting function that represents the evolution in time of the light distribution is the Gaussian function [15]. Nevertheless, in a confined gas flow, the light distribution may be affected by the combined mechanisms of streamwise advection and spanwise molecular diffusion in the direction perpendicular to the wall, thus differing from the Gaussian distribution in a quiescent gas. However, the image data revealed that these effects are negligible, and the Gaussian function is still representative of the light distribution in the gas flow direction. As it has been done in previous works [12,14,20], the Gaussian fitting applied to each horizontal line of pixels at any position $y=y_{j}$ is

(1) $$f\left(x\right)=\frac{a_{2}}{\sqrt{2\pi a_{3}}}e^{-\frac{{\left(x-a_{4}\right)}^{2}}{2a_{3}}}+a_{1},$$

where the fitting parameters $a_{1}$, $a_{2}$, $a_{3}$, and $a_{4}$ represent, respectively, the offset, the amplitude, the variance, and the peak position. Thus, $a_{4,j}$ corresponds to the displacement $s_{x,j}$ at each discrete location $y_{j}$. Figure 11 illustrates the procedure of the Gaussian fitting function applied to a horizontal line of pixels on an MTV image. As it can be observed, the statistical fluctuations characterizing the signal distribution on one horizontal line of pixels were significant with respect to the Gaussian peak. This certainly increased the uncertainty in the localization of the tagged line profile. Since the whole displacement data $s_{x,j}$ along the channel height will be afterwards fitted by the numerical solution $s_{x}\left(y_{j}\right)$ of the advection–diffusion equation, the uncertainty on the tagged line position at each location $y_{j}$ could be practically quantified by the ratio of the deviation between the displacement data and the numerical displacement profile, $s_{x,j}-s_{x}\left(y_{j}\right)$, to the value of the displacement profile $s_{x}\left(y_{j}\right)$ itself.

(f) Velocity reconstruction: the final step is the application of the velocity reconstruction algorithm to the set of displacement data $s_{x,j}$ determined in step (e). The reconstruction method of Frezzotti et al. [13] provides the velocity profile that makes the numerical displacement $s_{x}\left(y\right)$ fit at best the displacement dataset $s_{x,j}$. The numerical solving of the diffusion–advection equation requires providing the channel height H and the diffusion coefficient D characterizing the tracer diffusion through the background gas. The reconstruction method is sensitive to both parameters. For a given velocity profile u(y) and a given diffusion coefficient D, the displacement profile $s_{x}\left(y\right)$ at a certain time t has a thickness, defined as $\Delta s=\max s_{x}\left(y\right)-\min s_{x}\left(y\right)$, that is proportional to $H^{2}$ [13]. The value of the wall distance H used in defining the mathematical domain needs, therefore, to be as accurate as possible in order to have a correct velocity reconstruction. As discussed in step (b), the localization of the channel walls on the image cannot be done with the highest accuracy and is affected by possible imperfections in the calibration of the positioning of the channel and the laser beam with respect to the CCD. For flow regimes characterized by strong molecular diffusion, the numerical solution $s_{x}\left(y\right)$ is also sensitive to the value of the diffusion coefficient D.

## 5. CV Methodology and Data Processing

The local measurements from the molecular tagging are compared to global measurements obtained by means of the constant volume technique. The CV technique allows one to relate pressure variations with time to mass variation with time inside a reservoir of constant volume. Thus, monitoring the time evolution of the pressure in the upstream tank allows one to measure the mass flow rate $\dot{m}\left(t\right)$ at any time during the experimental run [21]. If the pressure variation due to thermal fluctuations is neglected, the equation relating pressure variation to mass variation is

(2) $${\dot{m}}_{CV}=-\frac{V}{R_{s}T}\frac{dp_{1}}{dt}.$$

where $R_{s}$ is the specific gas constant, which the value depends on the acetone concentration present in the gas mixture. The gas temperature T was assumed to correspond to the regulated temperature (24 °C) in the experimental room. The upstream reservoir corresponds to the region in Figure 2 encompassed by the dashed line, and it is composed of the two tanks and all valves and connection pipes that are upstream valve V1. The methodology used for measuring the volume V of the upstream reservoir consists of a simple thermodynamic experiment: (i) with valves V1 and V5 closed, the upstream reservoir is first vacuumed through valve V12 down to a pressure $p_{v}$; (ii) valve V12 is then closed and a small reference reservoir filled with air at atmospheric pressure $p_{atm}$ is connected to the vacuumed reservoir in correspondence of valve V12; and (iii) by opening valve V12, the air in the small reservoir is discharged into the upstream reservoir until an equilibrium pressure $p_{eq}$ is reached. The volume of the small reservoir is known to be $\bar{V}$ = 486.5 × 10^−6^ m^3^ and it has been accurately calculated by measuring its weight when filled with water. By considering that at the initial and final thermodynamic states the air is at ambient temperature, the volume of the reservoir is calculated from

(3) $$p_{v}V+p_{atm}\bar{V}=p_{eq}\left(V+\bar{V}\right).$$

This experiment has been repeated several times for a statistical characterization of the measurement uncertainty. The volume of the upstream reservoir has been measured to be V = 0.176 m^3^ with a relative uncertainty of 0.08%. The evaluation of the derivative $\frac{dp_{1}}{dt}$ is carried out by fitting the pressure data $p_{1}\left(t\right)$ with a proper analytical function. The most suitable fitting function for the pressure relaxation characterizing the type of experimental setup employed in this work is a series of N exponential functions:

(4) $$p_{1}=\overset{N}{\underset{i=1}{\sum }}a_{i}e^{-t/\tau_{i}}.$$

The choice of N is based on the length of the time interval of pressure data to be fit. For long time intervals, more exponential functions are used for obtaining the best fittings. For the experimental run of Figure 5, N = 4 provided an accurate fitting of the whole array of pressure data. Once the fitting parameters $a_{i}$ and $\tau_{i}$ have been determined, the rate of variation of the upstream pressure and the mass flow rate entering in the channel can be calculated.

**Figure 12:**
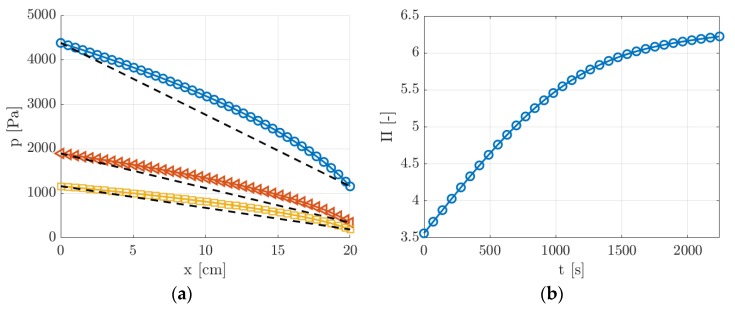
(**a**) Pressure distribution p(x,t) along the channel length at three different times t = 100, 1000, and 2000 s during the experimental run, which correspond to pressure ratios Π = 3.8 (O), 5.5 (◁), and 6.2 (**☐**). The dashed lines represent the linear pressure distributions when rarefaction and compressibility effects are neglected; (**b**) time evolution of pressure ratio Π during the experimental run.

From the measured inlet and outlet pressures $p_{1}$ and $p_{2}$, the axial pressure distribution p(x,t) along the channel can be predicted from the analytical solution obtained by Arkilic et al. [22]. Figure 12 reports p(x,t) at three different times t = 100, 1000, and 2000 s from the beginning of the experiment.

Since the pressure ratio $\Pi =p_{1}/p_{2}$ was relatively high, ranging from 3.5 to 6.5, the compressibility effects on the pressure distribution were not negligible for the whole duration of the experimental run. As shown in Figure 12b, even though the pressure difference $p_{1}-p_{2}$ decreased in time, the pressure ratio Π increased and, thus, the effects of the gas compressibility on the pressure distribution along the channel assumed even more importance at the lowest pressures. 

The mass flow rate ${\dot{m}}_{CV}\left(t\right)$ provided by the CV technique and the theoretical pressure distribution p(x,t) inferred from the pressure measurements $p_{1}\left(t\right)$ and $p_{2}\left(t\right)$ provided an estimation of the average velocity of the flow on the cross-section,$\;{\bar{u}}_{CV,2D}\left(x,t\right)$, at each position x along the channel and at each time t during the experimental run:

(5) $${\bar{u}}_{CV,2D}\left(x,t\right)=\frac{{\dot{m}}_{CV}\left(t\right)\;R_{s}T}{Hb\;p\left(x,t\right)}.$$

Nevertheless, ${\bar{u}}_{CV,2D}$ represents the average of the velocity over the entire cross-section of the channel, while the average velocity ${\bar{u}}_{MTV}$ measured by MTV represents the average value along a line at z = 0. Therefore, in order to have a meaningful comparison between MTV and CV data, the average value ${\bar{u}}_{CV,1D}$ of the velocity along this line was computed from the average value ${\bar{u}}_{CV,2D}$ on the section by using the analytical solution of the 2D velocity distribution over the cross-section provided by Ebert and Sparrow [19]. The analytical 2D velocity function was evaluated at z = 0 and averaged over the channel height, yielding the following relationship between ${\bar{u}}_{CV,1D}$ and ${\bar{u}}_{CV,2D}$:

(6) $${\bar{u}}_{CV,1D}\left(x,t\right)=K\left(x,t\right){\bar{u}}_{CV,2D}\left(x,t\right).$$

By setting the TMAC to 1, the value of the coefficient K(x,t) depends on the Knudsen number of the gas mixture and was therefore computed for each thermodynamic condition. Finally, the CV measurement that was compared to the average velocity ${\bar{u}}_{MTV}$ measured by MTV corresponded to the value of ${\bar{u}}_{CV,1D}\left(x,t\right)$ evaluated at x=L/2, which, for the sake of simplicity, will be just indicated as ${\bar{u}}_{CV}$. MTV measurements of the velocity profile might not fall exactly at z=0 along the channel width but the variations of the profile are less than 1% even 1 mm apart from the center. The comparison between MTV and CV velocity measurements would provide a validation and verification of the two experimental techniques and of the analytical solutions for the pressure distribution and for the 2D velocity profile of Ebert and Sparrow, which were both used for the calculation of ${\bar{u}}_{CV}$. 

Although the gas compressibility produces non-negligible variations of the flow properties along the channel length (Figure 12a), the investigated gas flow could always be assumed as locally fully developed, locally incompressible, and isothermal. The analytical solution for the pressure distribution provided by Arkilic et al. [22] can be employed only if these assumptions can be considered as valid. Harley et al. [23] compared the analytical solution based on these same hypotheses with the numerical solution of the full compressible Navier–Stokes equations for a slip gas flow in a microchannel. They reported discrepancies of less than 3% between the theoretical and numerical solutions of the axial velocity profile for local Mach numbers lower than 0.3. For the same range of Mach number values, the numerical results in [23] showed that the temperature drop due to the gas expansion along the channel is less than 3%. From the results of Equation (5) and by assuming an isothermal gas flow, the Mach number Ma(x,t) of the gas flow here investigated was calculated to be lower than 0.3 at any position along the channel length and at any time during an experimental run. 

## 6. Experimental Results

The presentation of the experimental data was organized in the following manner. Section 6.1 reported MTV measurements of argon flows in non-rarefied conditions and at pressures between 30 and 6 kPa. Section 6.2 presented MTV data of helium flow in rarefied conditions and at pressures between 1.6 kPa and 900 Pa. In Section 6.3, the comparison between MTV and CV data was analyzed for the cases presented in Section 6.1 and Section 6.2. The kinetic diameter for the acetone molecule used for all the calculations was d = 470 pm, as reported in Van der Perre et al. [24], which is also similar to the values reported in other recent works [25,26]. Moreover, since the investigated gas flow can be considered as isothermal, the mean free path of the mixture was estimated by modeling molecular interactions through hard sphere potentials.

### 6.1. MTV Measurements of Channel Gas Flows in Non-rarefied Conditions

In this section, the MTV technique was applied to acetone-seeded argon flows in non-rarefied conditions. The previous works of Samouda et al. [12] and Si-Hadj Mohand et al. [14] already demonstrated the successful application of MTV for velocity measurements in channel gas flow in non-rarefied conditions by using as a tracer acetone vapor excited at 266 nm. Si Hadj Mohand et al. were not able to apply MTV for pressures lower than about 42 kPa because of the low signal intensity due to the limited phosphorescence quantum yield of acetone excited at 266 nm. The results of this work were based on acetone vapor excited at 310 nm, which guaranteed a detectable and longer signal even for pressures lower than 1 kPa.

In order to demonstrate that the novel experimental setup provided accurate results in non-rarefied conditions, the first results presented in this work are for gas flows at pressures between 30 and 6 kPa. These are the first MTV results obtained for gas flows at pressures lower than 42 kPa. In these experiments, the mass flow rate, and thus the inlet over outlet pressure ratio were maintained low in order to make the upstream pressure vary slowly enough and to obtain quasistationary conditions during one experimental run. For the thermodynamic conditions considered in this section, the Mach number was at most 0.04, and the Knudsen number was not higher than 0.001. For this reason, the local compressibility and the rarefaction effects were negligible. In addition, the pressure variation along the channel was low (inlet–outlet pressure difference between 2% and 5% of the average pressure), and, consequently, the theoretical pressure distribution along the channel was quasilinear.

**Figure 13:**
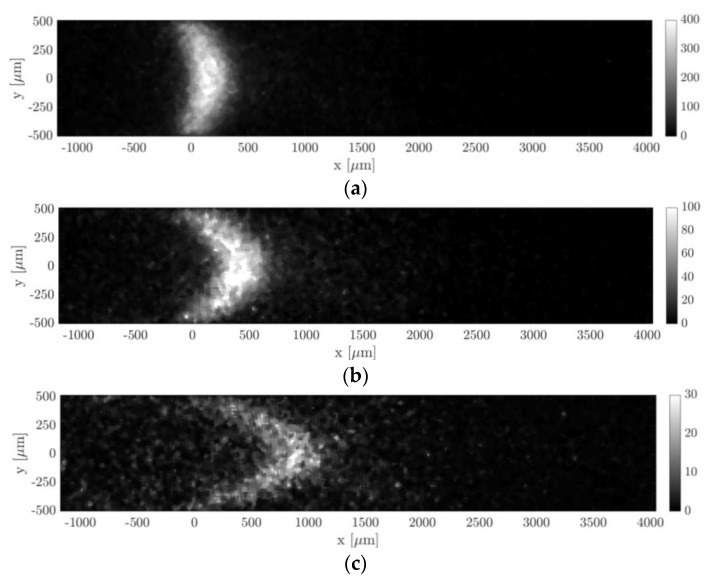
MTV acquisitions of argon–acetone flow with χ = 20%: (**a**) Δt = 10 µs, $p_{m}$ = 27.38 kPa, and Δp = 528.4 Pa; (**b**) Δt = 25 µs, $p_{m}$ = 28.83 kPa, and Δp = 538.8 Pa; and (**c**) Δt = 50 µs, $p_{m}$ = 30.36 kPa, and Δp = 549.9 Pa. The recording parameters are $N_{l}$ = 100, $N_{i}$ = 20, G = 100%, and $\Delta t_{gate}$ = 100 ns. The acquisitions are made with a 4 × 4 binning. The laser pulse energy was varying between 30 and 40 µJ.

**Figure 14:**
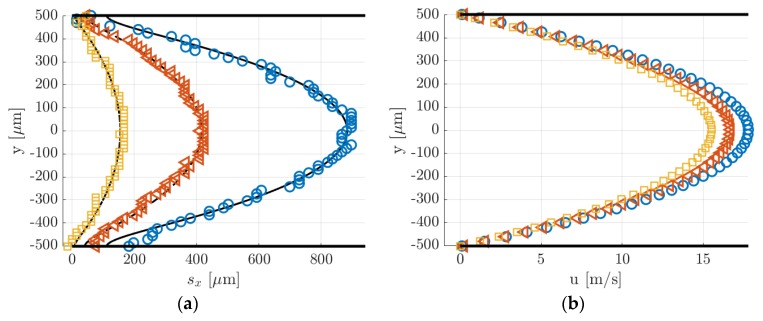
(**a**) Displacement data computed by applying the Gaussian fitting per line to the images of Figure 13. The black lines represent the numerical solutions for the displacement profile provided by the reconstruction method; (**b**) velocity reconstruction from the displacement data in (**a**): Δt = 10 µs (**☐**), 25 µs (◁), and 50 µs (O).

Figure 13 and Figure 14 show the molecular displacement of the tagged line through the channel, at different delays after laser excitation, from Δt = 10 to 50 µs. Each image represents the average of 20 images, each integrating 100 laser pulses. The acquisitions were carried out by setting the gain at its maximum value, i.e., G = 100%, the IRO gate at minimum, i.e., $\Delta t_{gate}$ = 100 ns, and by using a 4 × 4 binning. A 2D median filter was applied to each image for increasing its SNR. The average pressure and the inlet–outlet pressure difference were quite similar for images acquired at different delays Δt. Therefore, we considered that the comparison of the molecular displacement at different Δt was still representative of how the tagged line evolves as it moves through the channel. Average pressure $p_{m}$, inlet–outlet pressure difference Δp, mass flow rate ${\dot{m}}_{CV}$, Mach number Ma, and Knudsen number Kn that characterize each image are summarized in Table 1 and Table 2. As previously mentioned, since the experiments were conducted in quasistationary conditions, the thermodynamic conditions in the system varied with time. The tables reported the variations in percentage with respect to the average value of each property during 100 s, which is the time needed to acquire $N_{i}$ = 20 images.

#### 6.1.1. Experiments at High Pressure ($p_{m}$ = 29 kPa)

Hereafter we presented the results of an argon–acetone gas mixture with χ = 20% acetone molar concentration. The average mean pressure of the gas flow was $p_{m}$ = 28.85 kPa and the average Knudsen number was Kn = 1.75 × 10^−4^. Figure 13 shows how the molecular displacement of acetone inside the background gas evolved over time. Two physical phenomena are immediately recognizable in the images, which are the advection and diffusion of the tracer inside the gas flow. As the delay from the laser excitation increased, it is evident from the images how the signal intensity reduced.

In Figure 14a, the data points represent the displacement data $s_{x,j}$ obtained by applying to the raw images of Figure 13 the Gaussian fitting per line procedure described in Section 4. The application of the reconstruction method by Frezzotti et al. [13] to these data provides the velocity profiles shown in Figure 14b. The black lines reported in Figure 14a represent the numerical solutions provided by the advection–diffusion equation of the molecular displacement generated by the velocity profiles of Figure 14b. The reconstruction algorithm was successful in accurately extracting the distribution of the axial velocity. As expected, the slip velocity at the wall was very close to zero, with values ranging between 0.006 and 0.2 m/s. The diffusion coefficient D employed for the velocity reconstruction has been estimated by means of the Blanc’s law [15] and was set to 3.5 × 10^−5^, 3.3 × 10^−5^ and 3.2 × 10^−5^ m^2^/s, respectively, for the data at Δt = 10, 25, and 50 µs. Since argon and acetone have molecular mass and kinetic diameter of the same order of magnitude, small variations of the tracer concentration χ in different experimental runs did not modify considerably the estimated diffusion coefficient used in the reconstruction. Moreover, preliminary tests showed that for these experimental conditions the resulting velocity profile was quite insensitive to the value of the diffusion coefficient used.

#### 6.1.2. Experiments at Low Pressure ($p_{m}$ = 6.5 kPa)

**Figure 15:**
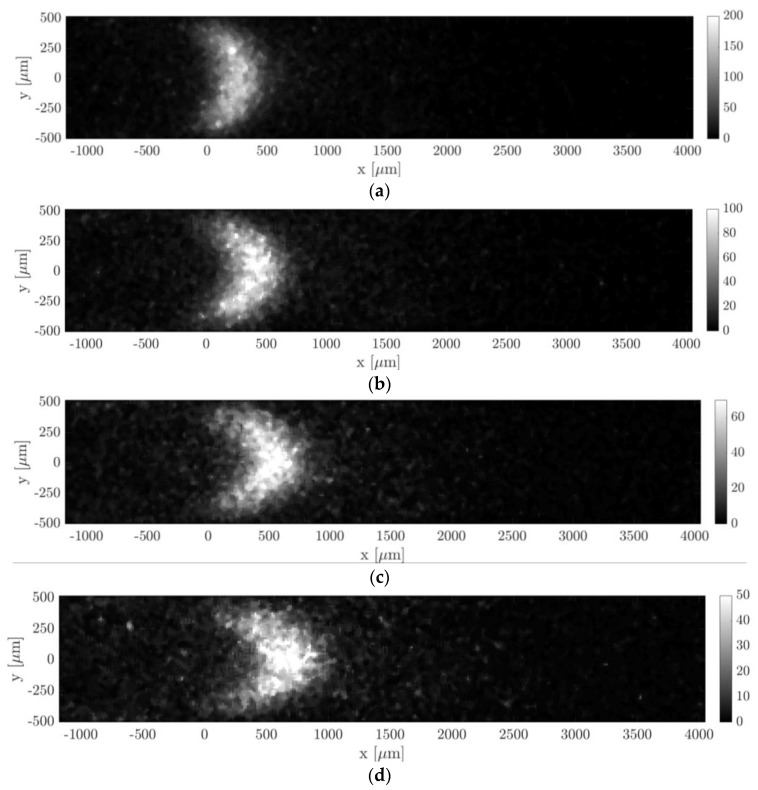
MTV acquisitions of argon–acetone flow with χ = 20%: (**a**) Δt = 20 µs, $p_{m}$ = 6904 Pa, and Δp = 371 Pa; (**b**) Δt = 30 µs, $p_{m}$ = 6620 Pa, and Δp = 367 Pa; (**c**) Δt = 40 µs, $p_{m}$ = 6337 Pa, and Δp = 362.8 Pa; and (**d**) Δt = 50 µs, $p_{m}$ = 6071 Pa, and Δp = 358.6 Pa. The recording parameters are: $N_{l}$ = 100, $N_{i}$ = 20, G = 100%, and $\Delta t_{gate}$ = 100 ns. The acquisitions are made with a 4 × 4 binning. The laser pulse energy was varying between 20 and 30 µJ.

Argon–acetone mixture flows were also investigated by MTV at lower pressures, in order to verify the reconstruction method in non-rarefied conditions but with higher molecular diffusion. The images of Figure 15 illustrate MTV acquisitions carried out at an average pressure of 6.5 kPa and at Δt = 20, 30, 40, and 50 µs. All thermodynamic characteristics of the observed experimental flow are reported in Table 2. As for the case at higher pressure, the thermodynamic properties associated to each image were not exactly the same but, again, quite similar. Since the gas pressure was 4 times lower than the case presented previously, the Knudsen number was 4 times higher.

**Figure 16:**
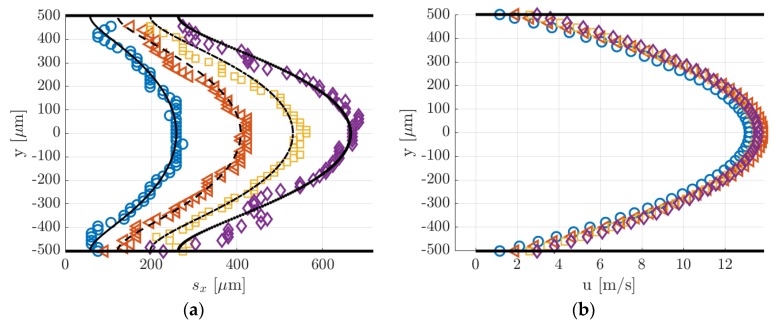
(**a**) Displacement data computed by applying the Gaussian fitting per line to the images of Figure 15. The black lines represent the numerical solutions for the displacement profile provided by the reconstruction method; (**b**) velocity reconstruction from the displacement data in (**a**): Δt = 20 µs (O), 30 µs (◁), 40 µs (**☐**), and 50 µs (**◇**).

Figure 16a,b reports, respectively, the displacement data with the corresponding numerical displacement profiles and the reconstructed velocity profiles. The diffusion coefficients used for the reconstructions were 1.39 × 10^−4^, 1.46 × 10^−4^, 1.52 × 10^−4^, and 1.59 × 10^−4^ m^2^/s, respectively, for the data at Δt = 20, 30, 40, and 50 µs. As it can be observed, the reconstruction method was not able to provide here an accurate velocity profile. The slip velocity at the wall, which was expected to be limited to a maximum of 0.05 m/s, ranged between 1 and 2.6 m/s, depending on the delay of the displacement data used. Figure 16a shows that the displacement data were well fitted by the numerical solution provided by the reconstruction method. Except for the case at Δt = 50 µs, the dispersion of displacement data around the numerical solution was quite low. However, while the relatively high SNR at the channel centerline y = 0 made the identification of the deformed tagged line quite accurate, there might be an uncertainty on the local displacement close to the wall. With respect to the displacement profiles at 28 kPa previously reported, the molecular displacement at the wall was, in this case, definitely larger as a consequence of the higher molecular diffusion. The accurate evaluation of the molecular slip at the wall appeared to be of fundamental importance for extracting a correct velocity profile.

### 6.2. MTV Measurements of Channel Gas Flows in Rarefied Conditions

**Figure 17:**
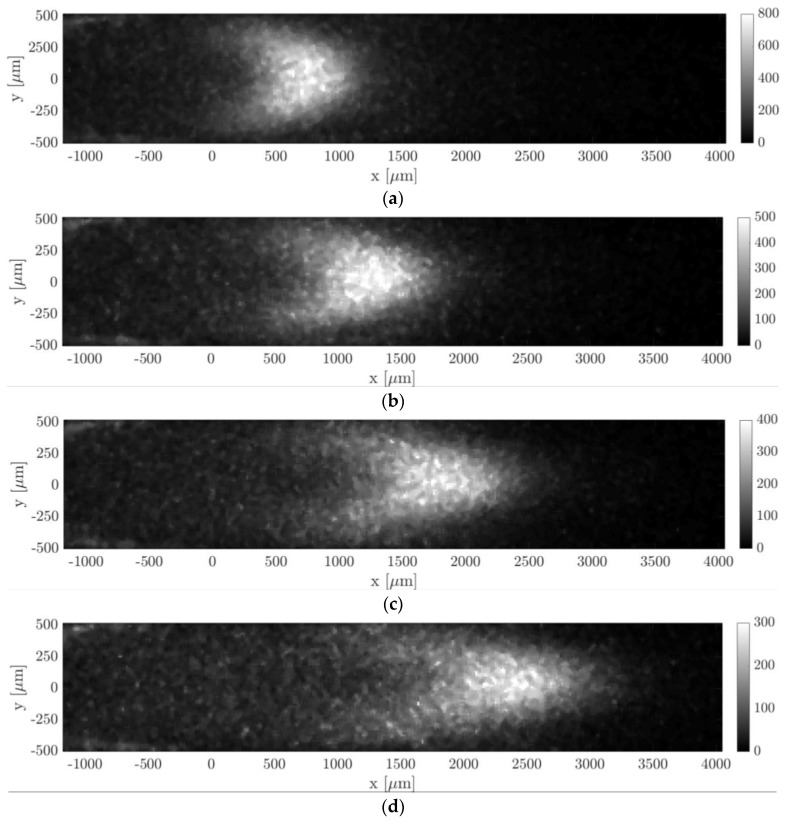
MTV acquisitions of helium–acetone flow with χ = 20%: (**a**) Δt = 20 µs, $p_{m}$ = 1576 Pa, and Δp = 1789.2 Pa; (**b**) Δt = 30 µs, $p_{m}$ = 1580 Pa, and Δp = 1793.2 Pa; (**c**) Δt = 40 µs, $p_{m}$ = 1576.6 Pa, and Δp = 1789.2 Pa; and (**d**) Δt = 50 µs, $p_{m}$ = 1578.7 Pa, and Δp = 1791.8 Pa. The recording parameters used are: $N_{l}$ = 100, $N_{i}$ = 20, G = 100%, and $\Delta t_{gate}$ = 500 ns. The acquisitions are made with a 4 × 4 binning. The laser pulse energy was varying between 80 and 120 µJ.

**Figure 18:**
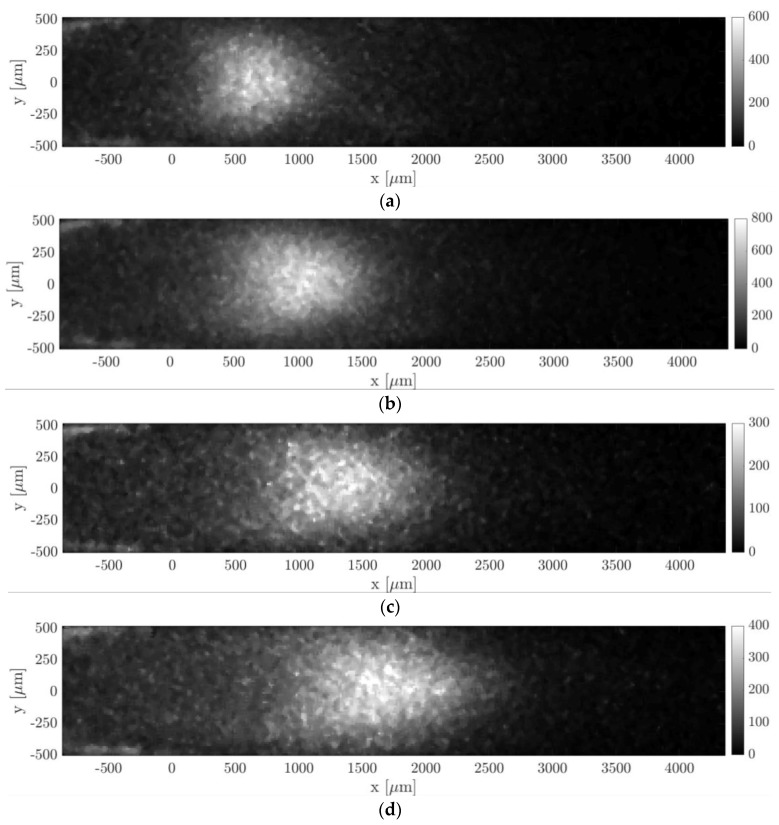
MTV acquisitions of helium–acetone flow with χ = 20%: (**a**) Δt = 20 µs, $\Delta t_{gate}=$ 500 ns, $p_{m}$ = 920 Pa, and Δp = 1092.7 Pa; (**b**) Δt = 30 µs, $\Delta t_{gate}$ = 1000 ns, $p_{m}$ = 920.3 Pa, and Δp = 1093.4 Pa; (**c**) Δt = 40 µs, $\Delta t_{gate}$ = 500 ns, $p_{m}$ = 919.1 Pa, and Δp = 1091.8 Pa; and (**d**) Δt = 50 µs, $\Delta t_{gate}$ = 1000 ns, $p_{m}$ = 919.1 Pa, and Δp = 1090.7 Pa. The recording parameters used are: $N_{l}$ = 100, $N_{i}$ = 20, and G = 100%. The acquisitions are made with a 4 × 4 binning. The laser pulse energy was varying between 80 and 100 µJ.

In this section, MTV was applied to helium–acetone mixture flow with χ = 20% in the slip regime. Two experimental conditions of this gas flow were here investigated. The first one was at an average pressure of about 1.6 kPa, the second one was at 920 Pa. All properties of the visualized gas flow are reported in Table 3 and Table 4, along with their range of values that characterized the images used for averaging. Figure 17 and Figure 18 illustrate the tracer displacement, respectively for the gas flow at 1.6 kPa and at 920 Pa, for four different delays after the laser excitation, at Δt = 20, 30, 40, and 50 µs. These images are the first flow visualizations ever obtained in confined and rarefied gas flows. They represent the experimental demonstration of the molecular displacement in a channel theoretically predicted by the diffusion–advection equation [13]. Due to the high repeatability of the experimental flow that the gas circuit can guarantee at low pressures, the four images corresponded to the very same average thermodynamic conditions. The Mach and the Knudsen numbers were on average, respectively, Ma = 0.07 and Kn = 0.008 for the case at 1.6 kPa, and Ma = 0.045 and Kn = 0.014 for the case at 920 Pa. The two investigated gas flows could then be considered as locally incompressible and at the beginning of the slip flow regime. Even though the Knudsen number of the gas flow at 920 Pa was almost twice as much as at 1.6 kPa, the slip velocity at the wall was estimated to be almost the same, 2.2 m/s and 2 m/s, respectively. This is because the flow speed imposed by the vacuum pumps decreased as the working pressure decreased.

The pressure difference and mass flow rate were, respectively, 1790 Pa and 1.7 × 10^−6^ kg/s for the images of Figure 17, and 1092 Pa and 1.7 × 10^−7^ kg/s for the images of Figure 18. The flow rate of these flows was higher than the one characterizing the argon–acetone flows, as it can be noted by comparing the imaged molecular displacements of Figure 17 and Figure 18 with those of Figure 14 and Figure 16. As for the previous case of argon–acetone flow, each image of Figure 17 and Figure 18 represents the average of $N_{i}$ = 20 images. However, because of the higher flow rates, the relative variations of the thermodynamic properties associated to a group of $N_{i}$ averaged images were higher than for the data of argon flows. Nevertheless, even though the mass flow rate varied up to 6.5%, the variations on the velocity profile were limited to less than 3%, as previously discussed in Section 3. 

In all images of Figure 17 and Figure 18, a peculiar noise was observed behind the wake of the moving emitting molecules, where the origin was still unclear. It is possible that this emission was provided by tagged molecules that have diffused from the center towards the lateral walls of the channel, where the gas velocity slows down.

By comparing the images of Figure 17 and Figure 18 with those of Figure 13 and Figure 15, it can be observed that the light emission was stronger for the data related to acetone–helium flow, even though the gas pressure was lower. This is because the IRO gate $\Delta t_{gate}$ was increased and the average energy of the laser excitation was raised to values between 80 and 120 µJ. For the case at 1.6 kPa, $\Delta t_{gate}$ was set to 500 ns, while for the gas flow at 920 Pa, IRO gates of 500 ns and 1000 ns were both used to investigate whether this acquisition parameter could improve or not the SNR of the MTV images. In particular, $\Delta t_{gate}$ = 500 ns was used for the images at Δt = 20 and 40 µs, whereas $\Delta t_{gate}$ = 1000 ns was used for the images at Δt = 30 and 50 µs. Doubling the IRO gate doubled the amount of light collected on the CCD, as expected, but it did not seem to drastically improve the SNR of the acquisition.

The images of Figure 18 anticipate which are the difficulties that will be encountered in applying MTV to gas flows at pressures even lower than those here investigated. As the working pressure decreased, the highest mass flow rate imposed by the two downstream vacuum pumps decreased as well. The reduced gas flow speed combined with the increased molecular diffusion makes the Taylor dispersion become very strong, which significantly flattens the profile of the tagged line. The increasing strength of the Taylor dispersion can already be noticed by comparing the MTV images of Figure 17 with those of Figure 18. At 1.6 kPa, the tagged line was deformed in time by the gas movement, and despite the relatively high molecular diffusion the CCD sensor could still clearly display the shape of the displacement profile. For the gas flow at 920 Pa, the Taylor dispersion and the molecular diffusion were starting to dominate over the gas advection and the tagged line was tending to become a cloud of molecules that migrated in the flow direction without preserving a defined shape. The current MTV setup was able to resolve the displacement profiles even at pressures as low as 920 Pa, but it might and probably failed in providing local information on the gas molecular displacement in flows at lower pressures. However, this limitation of the current experimental setup could be overcome by (i) increasing the CCD resolution, e.g., by reducing the binning or by increasing the magnification of the lenses system, and/or by (ii) adding more vacuum pumps at the channel outlet. The former solution would allow us to resolve smaller deformations of the tagged line, but it would have the drawback of reducing the amount of light collected and the SNR of the image data. The latter option would, instead, increase the speed of the gas flow thus decreasing the effects of the Taylor dispersion on the displacement profile. Increasing the flow speed would also favorably increase the magnitude of the slip velocity at the wall characterizing the observed gas flow. Nevertheless, in order to maintain the gas flow in a local-incompressibility regime, the Mach number needs to be kept low enough, and so the downstream pumping force cannot be raised too much.

**Figure 19:**
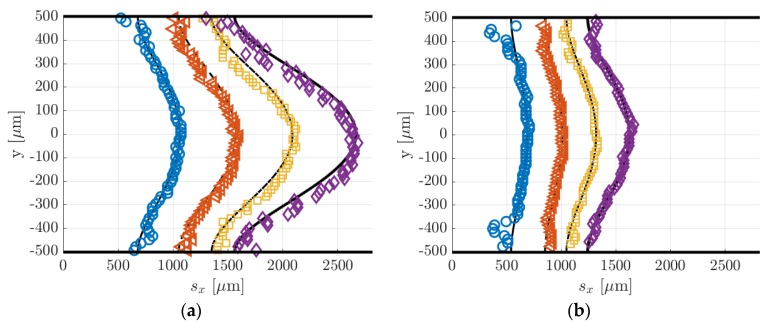
(**a**) Displacement data computed by applying the Gaussian fitting per line to the MTV images at (**a**) $p_{m}$ = 1580 Pa (Figure 17) and (**b**) $p_{m}$ = 920 Pa (Figure 18): Δt = 20 µs (O), 30 µs (◁), 40 µs (**☐**), and 50 µs (**◇**). The black lines represent the numerical solutions for the displacement profile provided by the reconstruction method. The two figures are on the same spatial scale.

Figure 19 reports the displacement data along with the numerical displacement profiles that result from the application of the reconstruction method. Since the displacement data at different delays corresponded to the same thermodynamic conditions, the diffusion coefficient D used in the reconstruction method was the same for the four sets of displacement data and was calculated by means of the Blanc’s law [15] to be 1.44 × 10^−3^ m^2^/s for the data at 1.6 kPa and 2.47 × 10^−3^ m^2^/s for the data at 920 Pa. Once again, the reconstruction method failed in extracting the correct velocity profile from the displacement data. The same issue encountered in the velocity reconstruction from the displacement data of argon–acetone flow at 6.5 kPa (Figure 16) appeared here in an even more dramatic way. The reconstructed slip velocity ranged between −16 and +20 m/s, while the expected analytical values should be around 2 m/s, when Maxwell boundary conditions were employed, and full accommodation of the momentum was assumed. The velocity profiles that result from the application of the reconstruction method to the displacement data of Figure 19 were not here presented because qualitatively identical to those reported in Figure 16b, while differing only for the higher fluctuations on the slip velocity.

For a given set of parameters, the reconstruction method provides the velocity profile that generates the molecular displacement that best fits the displacement data points. For this reason, despite the errors on the velocity profiles, the reconstructed displacement profiles (black lines in Figure 18a) always followed very well the displacement data. The inaccuracies on the reconstructed local velocity were likely due to inaccuracies on the height H and diffusion coefficient D used in the reconstruction algorithm (see [13] for more details on the reconstruction method). The reconstructed method is quite sensitive to these parameters, and its sensitivity increases as the molecular slip at the wall gains importance. For the argon–acetone flow at 28 kPa, the sensitivity of the reconstruction to H and D was much lower because of the limited molecular slip at the wall by Taylor dispersion. For this reason, although the inaccuracy on H and D was the same for every experiment, the reconstruction method provided more accurate velocity profiles at high pressures.

Despite the inefficacy of the current reconstruction algorithm in extracting accurate information on the local velocity, the sets of displacement data reported in Figure 19a,b appeared to be qualitatively very good, in the sense that the data dispersion around the reconstructed displacement profiles was very limited. Especially for the data of Figure 19b, despite the stronger Taylor dispersion that increased the molecular slip at the wall and flattened the displacement profile, the current MTV setup was still able to capture accurate information on the gas molecular displacement even in gas flows at pressures as low as 920 Pa. Relatively large fluctuations of the displacement data were only encountered for the case at 920 Pa and at Δt = 20 µs in regions close to the walls. This is because the corresponding MTV image (Figure 18a) was somehow characterized by a quite low SNR in those regions, where the Gaussian fitting per line failed to identify the correct position of the tagged line. 

The displacement profiles of Figure 19a,b were represented on the same spatial scale to allow an easier comparison between the two cases. It was evident how the displacement profiles at 920 Pa had a much lower thickness, which is defined as $\Delta s_{x}\left(t\right)=\max\;\left(s_{x}\left(y\right)\right)-\min\;\left(s_{x}\left(y\right)\right)$ [15], as a result of the increased Taylor dispersion. In comparison to the displacement profiles related to argon–acetone flows of Figure 14a and Figure 16a, the displacement profiles in Figure 18a had a larger thickness. Even though the higher molecular diffusion tended to make the displacement profile flatter, the helium flow at 1.6 kPa was characterized by a speed that was about 4 times higher than the argon flow speed, thus making the molecular advection more pronounced. However, for the helium–acetone flow at 920 Pa, the molecular diffusion was so strong that, even though its flow speed was about 3 times that of the argon–acetone flow at 6.5 kPa, the measured displacement thickness resulted in being of the same order of magnitude of that measured in argon flow. Table 5 reports the values of the thickness $\Delta s_{x}$ related to the data of helium–acetone flows and the data of argon–acetone flow at 6.5 kPa.

### 6.3. Comparison between MTV and CV Data

In this section, MTV and CV techniques were cross-validated by comparison of the measured average velocity at the test section. Since the average velocity ${\bar{u}}_{CV}$ is derived from the mass flow rate data through analytical formulas, the following data comparison represents also a validation of the theoretical models of the pressure distribution along the channel length of Arkilic et al. [22] and the velocity distribution across the channel section of Ebert and Sparrow [19]. In order to make the data comparison more meaningful, the results were also provided with estimated uncertainty values. The uncertainty ranges reported in Table 6, Table 7, and Table 8 correspond to ± 2 standard deviations, with an associated confidence interval of 95%.

For the CV measurements, the uncertainty on ${\bar{u}}_{CV}$ was determined by the uncertainty on the average velocity ${\bar{u}}_{CV,2D}$ over the cross-section. Using Equation (2) in Equation (5) yields

(7) $${\bar{u}}_{CV,2D}=-\frac{V}{Hb\;p_{m}}\frac{dp_{1}}{dt},$$

which shows how ${\bar{u}}_{CV,2D}$ depends neither on the gas temperature T in the upstream reservoir nor on the acetone concentration χ composing the gas mixture flowing through the channel. The main uncertainty was given by the channel height, which has been measured by CCD imaging of the Suprasil^®^ windows re-emission. The uncertainty on H was, therefore, estimated based on the CCD resolution, and, because the employed measurement technique has limited accuracy, was conservatively estimated to be twice a pixel size on the CCD. A relative standard deviation on ${\bar{u}}_{CV}$ of 3% was computed by Taylor expansion of Equation (7) and by assuming that all parameters of the equation are statistically uncorrelated [27].

For the MTV measurements, the average velocity ${\bar{u}}_{MTV}$ was computed by averaging the reconstructed velocity profiles over the height. Although the local velocity results provided by the reconstruction method depend on the accuracy of the height H and the diffusion coefficient D used in the algorithm, the average velocity ${\bar{u}}_{MTV}$ only depends on the measured mean displacement ${\bar{s}}_{x}$ and the delay Δt. This explained why, even though the MTV velocity profiles might be inaccurate, the reconstruction method always provided profiles with an average velocity that is identical to an average velocity computed simply as

(8) $${\bar{u}}_{MTV}=\frac{{\bar{s}}_{x}}{\Delta t}.$$

As for Equation (7), ${\bar{s}}_{x}$ and Δt are assumed to be statistically uncorrelated. The mean displacement ${\bar{s}}_{x}$ was calculated from the displacement profiles $s_{x}\left(y\right)$ provided by the reconstruction method. For most of the experimental measurements presented in Section 6.1 and Section 6.2, the fluctuations of the displacement data point $s_{x,j}$ around the numerical solution $s_{x}\left(y\right)$ were relatively limited; therefore the computation of ${\bar{s}}_{x}$ from the displacement data practically provided the same results. When the data fluctuation is stronger, e.g., as for the data at Δt = 20 s in Figure 19b, using the reconstructed displacement profile for the computation of ${\bar{s}}_{x}$ helps in filtering the outlier data out. The delay Δt was known with an accuracy of the order of 5 ns, that is the precision of the PTU in synchronizing the triggering signals. Thus, for the lowest delays of acquisition employed, the maximum relative uncertainty on Δt was only 0.05%. Therefore, since the uncertainty on Δt was negligible, the accuracy on the average velocity ${\bar{u}}_{MTV}$ was solely determined by the accuracy on ${\bar{s}}_{x}$. Uncertainties on the measured mean displacement were introduced by the limited CCD resolution and by the low SNR of the image. The magnitude of the mean displacement determines whether the uncertainty on ${\bar{s}}_{x}$ is dominated by the former or the latter type of errors. The estimation of the uncertainty due to low SNR was, however, a difficult task, and more MTV data were required for its quantification. Therefore, a standard deviation of 1 pixel size, which is 15.2 µm, was associated to the measurement of ${\bar{s}}_{x}$ with the awareness of possibly underestimating it.

#### 6.3.1. Experiments in Argon–acetone Flow at 28 kPa

Table 6 reports the average velocities measured by MTV and the CV technique, with the corresponding uncertainty ranges. The relative error between MTV and CV measurements is also reported. Especially at high delays, the comparison demonstrated a good match between ${\bar{u}}_{CV}$ and ${\bar{u}}_{MTV}$. The mean displacements at Δt = 10, 25, and 50 µs were, respectively, ${\bar{s}}_{x}$ = 103.87, 280.97, and 598.56 µm, and thus the corresponding relative standard deviations on ${\bar{u}}_{MTV}$ due to the CCD spatial resolution were 14.6%, 5.4%, and 2.5%. The small variations on the measured values of ${\bar{u}}_{CV}$ followed the variations on the inlet–outlet pressure difference characterizing the average MTV image (see Table 1). At Δt = 10 µs, the SNR of the image (Figure 14a) was relatively high, but the mean displacement was about 7 pixels only, which explained the relatively high discrepancy with respect to the CV measurement. In this case, the CCD resolution dominated the uncertainty on ${\bar{s}}_{x}$, thus the estimated error range on ${\bar{u}}_{MTV}$ was most likely correct. This was also suggested by the fact that the relative deviation between MTV and CV measurements decreased as a function of the delay. At higher delays, however, the uncertainty range might be underestimated, since the SNR of the image visibly was reduced (Figure 14c). 

#### 6.3.2. Experiments in Argon–acetone Flow at 6.5 kPa

Despite the inaccuracy on the reconstructed velocity profile, the average velocity ${\bar{u}}_{MTV}$ was very close to the values ${\bar{u}}_{CV}$ calculated from the mass flow rate measurements (Table 7). As for the case at higher pressure, higher delay times provide a better match of the two measurements. The measured mean displacements were ${\bar{s}}_{x}$ = 181.4, 294.8, 396.5, and 494.6 µm, and the resulting relative standard deviations on ${\bar{u}}_{MTV}$ were 8.4%, 5%, 3.8%, and 3%, respectively for Δt = 20, 30, 40, and 50 µs. The Taylor dispersion amplified the molecular slip at the wall, which contributed more to the resulting mean displacement. Therefore, the imprecision in capturing the molecular slip at the wall contributed more to increase the uncertainty on the average velocity. Nevertheless, the SNR of the images of Figure 15 was relatively high, and the measured ${\bar{u}}_{MTV}$ differed from the values ${\bar{u}}_{CV}$ by less than 3% at Δt = 30 µs and only by about 0.5% at Δt = 40 and 50 µs. The variations on ${\bar{u}}_{CV}$ measured at different delays were limited to about 3% and reflected the slight decrease on the pressure difference characterizing the MTV images (see Table 2). The decreasing relative deviation between CV and MTV measurements indicates that the variations on ${\bar{u}}_{MTV}$ measured at different delays were mainly determined by the inaccuracies on the measured molecular displacement due to the CCD resolution. 

#### 6.3.3. Experiments in Helium–acetone Flows

As for the case of argon–acetone flow, even though the reconstructed velocity was inaccurate, the average velocity was well extracted from the displacement data and was always approximately the same, regardless of the delay considered. Table 8 and Table 9 compared the values of ${\bar{u}}_{MTV}$ with those of ${\bar{u}}_{CV}$. Since the helium–acetone flow was characterized by much higher mean molecular displacements, the relative standard deviation on ${\bar{u}}_{MTV}$ given by the CCD resolution was at most 1.7%, for the data at 1.6 kPa, and 2.4%, for the data at 920 Pa. 

The highest values of ${\bar{s}}_{x}$ were recorded for the helium flow at 1.6 kPa, where it varied from 0.89 mm at Δt = 20 µs to 2.15 mm at Δt = 50 µs, which was more than 4 times the displacement imaged in the argon–acetone flows. Even for the helium flow at 920 Pa, the measured mean displacements were relatively high: ${\bar{s}}_{x}$ = 620.22, 936.58, 1192.21, and 1444.39 µm, respectively for Δt = 20, 30, 40, and 50 µs. Therefore, the uncertainty on ${\bar{u}}_{MTV}$ was likely dominated by the SNR of the images. The molecular displacement at the wall was large and comparable to the centerline displacement, and therefore it contributed considerably to the measured ${\bar{s}}_{x}$. Since the SNR was lower in regions close to the channel walls, inaccuracies in measuring the displacement slip at the wall might bias the measured mean displacement. Moreover, the high molecular diffusion makes the tagged line thicker, which reduces the accuracy of the Gaussian fitting procedure in localizing the peak position. These considerations suggest that the uncertainties for ${\bar{u}}_{MTV}$ reported in Table 8 and Table 9 were probably underestimated. For the case at 920 Pa, the molecular slip at the wall represented between 85% and 90% of the mean molecular displacement. This might explain why the difference between CV and MTV measurements were higher than for the case at 1.6 kPa, where the molecular slip at the wall contributed to the mean displacement at about 75%. However, the 4 values of ${\bar{u}}_{MTV}$ were measured by repeating the experiment 4 different times, and the MTV velocity measured at different delays varied around the average value of 2% for the data at 1.6 kPa and 4.5% for the data at 920 Pa. These variations were of the same order of magnitude of the estimated variations due to fluctuations of the thermodynamic conditions characterizing the 20 averaged images (Section 6.2). As it can be noted from Table 8 and Table 9, the value of ${\bar{u}}_{MTV}$ was always higher than that of ${\bar{u}}_{CV}$, and their relative deviation was on average about 6.2% for the flow at 1.6 kPa and 11.6% for the flow at 920 Pa. This suggests the existence of a constant biasing error affecting the measurements. While MTV measurements are only based on the optical tracking of the gas displacement, the calculation of the CV average velocity makes use of the measured dimensions of the channel cross-section. At working pressures of the order of 1 kPa, the distance between the Suprasil^®^ windows might be slightly reduced by the higher external pressure. Therefore, the bias between CV and MTV average velocities was probably partly due to the uncertainty on the value of H. The idea that the compression of the channel height should be higher at lower pressures was reflected by the higher differences between CV and MTV data obtained for the case at 920 Pa. Variations of 6.2% and 11% on the channel height could correspond to a displacement towards the interior of the channel of only 30 µm and 55 µm, respectively, for each Suprasil^®^ window. In addition, if the windows were no longer perfectly aligned with the channel walls, this misalignment could create local velocity perturbation that affect the measured displacement profile.

**Figure 20:**
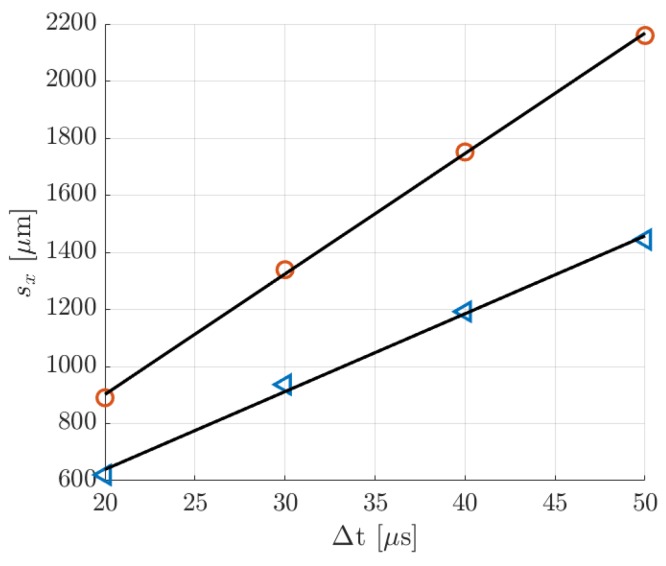
Mean molecular displacement ${\bar{s}}_{x}$ in helium flow at delays Δt = 20, 30, 40, and 50 µs: (O) data at 1.6 kPa; (◁) data at 920 Pa. The black solid line represents the linear interpolation of the MTV data, which provides an average velocity ${\bar{u}}_{MTV}$ = 42.2 m/s for the data at 1.6 kPa and ${\bar{u}}_{MTV}$ = 27.28 m/s for the data at 920 Pa.

Since the images of Figure 17 and Figure 18 represent the very same flow conditions and, thus, the same velocity profile, the mean displacement of the tagged lines evolved linearly with time, as demonstrated in Figure 20. 

A linear interpolation of these data provided a second measurement for the average velocity. For the case at 1.6 kPa, this second measurement was $\bar{u}{{}^{\prime }}_{MTV}$ = 42.2 m/s, which differed by about 4% from the average of the reconstructed velocity profiles, and only 2.3% from the average velocity measured by the CV technique. For the flow at 920 Pa, $\bar{u}{{}^{\prime }}_{MTV}$ = 27.28 m/s, which was smaller than the average of the reconstructed velocity profiles by 10% but differed only by 2.2% from the CV value. The MTV velocity that resulted from the linear fitting was likely more accurate than the average velocities measured from each MTV image individually, since more displacement data were used at once. This final result suggests the idea that the reconstruction algorithm might provide more accurate velocity profiles if properly modified to allow processing all the displacement data at different delays simultaneously. 

## 7. Discussions and Conclusions

This work demonstrated a successful application of the MTV technique to low-pressure gas flows in a millimetric channel. To the best of our knowledge, the images of Figure 17 and Figure 18 are the first flow visualizations ever reported of a gas flow in the slip regime and in a confined domain. MTV data were reported for gas flows with Knudsen numbers as high as Kn = 0.014. For the case of helium–acetone flow, a slip velocity at the wall of about 2 m/s is expected when a Maxwell boundary condition is used, which indicates that rarefaction has significant effects at the wall in the investigated flow conditions. As shown in Figure 14a, Figure 16a and Figure 18a,b, MTV could provide, even at pressures as low as 920 Pa, accurate data on the gas displacement, which was very well fitted by the numerical prediction of the 1D advection–diffusion equation. In particular, the MTV data here reported gave clear experimental evidences of the strong effects produced by the Taylor dispersion on the molecular displacement, which was, up to now, only theoretically and numerically predicted [13,15]. The comparison with the CV technique demonstrated also the good accuracy of the MTV technique in providing average displacement and average velocity measurements in rarefied gas flows. Some estimations of the uncertainties on the measured velocities were provided. A better quantification of the errors on the MTV average velocity would require the collection of more experimental data, thus allowing a more complete statistical analysis of the fluctuations on the signal generated by the molecular diffusion, the phosphorescence emission, and the intensified CCD itself. However, as demonstrated in Section 6.2, repeating the same experiments several times and using different delays of acquisition provided substantially the same MTV average velocity, which strongly supported the conclusion that the MTV measurements of the gas molecular displacement were characterized by a good precision, even for the highest Knudsen numbers investigated.

The success of our MTV implementation to the case of low-pressure gas flows in a confined domain was the result of two important experimental achievements. Firstly, an intense phosphorescence signal at pressures of the order of 1 kPa could be obtained only by selecting the optimal wavelength for the acetone excitation, which was 310 nm. In our previous work [15], we demonstrated that this laser wavelength allowed increasing the phosphorescence signal of at least 10 times with respect to an excitation at 266 nm. The second key aspect that made MTV application in confined and low-pressure gas flows possible is the open loop configuration chosen for the gas flow system. Actually, an open loop gas circuit is, for the current version of the tested channel, the only practical option for having an oxygen-free gas mixture at the test section. The tested channel used for MTV measurements needs to guarantee optical accesses for both laser excitation and CCD acquisition. These requirements make the channel be an assembly of different materials, which easily introduces leakages when used in a low-pressure system. This can generate a small flow of oxygen molecules, especially in correspondence of the Suprasil^®^ windows, which tends to quench the tagged acetone molecules. The current open loop configuration overcomes this issue by feeding the test section with fresh acetone–gas mixture at relatively high flow rates. Future improvements of this work envisage a better design of the test channel that aims to make it more leakage-proof. This will further improve the SNR of MTV images and might allow MTV applications even in a closed loop gas circuit, which has the advantage over the open loop version of providing more stable thermodynamic conditions for MTV acquisitions.

In non-rarefied flows and for pressure conditions at which the effects of the molecular diffusion on the evolution of the molecular displacement profile were limited, the reconstruction method could provide accurate local velocity measurements along the channel height and could correctly predict a negligible slip velocity at the wall. However, when the gas pressure was low enough, the Taylor dispersion significantly distorted the tagged line, and thus the current version of the reconstruction method was unable to extract accurate information on the local gas velocity, despite the very good match between MTV and CV measurements and the relatively high quality of the MTV images and of the measured displacement profiles. Further investigation is necessary to understand whether the available MTV dataset is not large enough or the post-processing method needs to be improved (or both).

Preliminary analysis revealed a significant sensitivity of the reconstructed velocity profile to the parameters H and D. Future efforts aim to better characterize the sensitivity of the post-processing algorithm and to improve the accuracy on the measurements of the channel height and of the diffusion coefficient. An important challenge related to the latter intent is given by the fact that the diffusion coefficient D strongly depends on the value used for the acetone molecular diameter d. In this work, the value d = 470 pm provided by Van der Perre et al. [24] was considered because this result is supported by several other authors [25,26]. Nevertheless, there are also other works in the literature that provide quite different estimations of the acetone kinetic diameter, which allow variations from 460 to 730 pm (590 pm by Almy et al. [28], 616 pm in Nydatok et al. [29], and 730 pm in Frezzotti et al. [13]). These uncertainties on the molecular diameter introduce relative uncertainties on the diffusion coefficient D that can be as large as 100%. New experimental data on d and D would be, therefore, of great interest for the improvement of this work. Differently from the parameter D, channel height H can be directly measured, thus the uncertainty on this parameter can be highly reduced. Currently, the accuracy on H is mainly affected by the difficulty in controlling the Suprasil^®^ windows position, which can slightly change as a function of the working gas pressure. Future new designs of the channel test section will aim to solve this issue, thus drastically improving the accuracy on the measurement of H. Nevertheless, since the displacement profile solution of the advection–diffusion equation depends on $H^{2}$ [13], the reconstruction method is much more sensitive to H than to D. In this context, new versions of the velocity reconstruction algorithm that are more robust to uncertainties on the parameters H and D are under development.

Furthermore, it is also plausible that the advection–diffusion equation used for the velocity reconstruction does not capture all the physical features of the pressure-driven binary gas flow. For instance, the high molecular mass ratio of the helium–acetone mixture and the relatively strong pressure gradient driving the investigated gas flow might generate a velocity difference between the two species, thus leading to an effect known as gas separation [30]. Future efforts will also aim to investigate (i) if this phenomenon introduces a non-negligible velocity bias between helium and acetone molecules and (ii) if it is a key mechanism that needs to be embedded in the reconstruction algorithm.

In conclusion, despite the new challenges encountered in carrying out local velocity measurements in gas flows characterized by high molecular diffusion, this work demonstrated that MTV is currently the most promising technique for providing direct measurements of the slip velocity at the wall in rarefied gas flows.

## Figures and Tables

**Table 1:** Flow properties associated to the images of Figure 13. The reported percentage change indicates how much the properties vary inside the group of $N_{i}$ = 20 averaged images.

Image	Δt (µs)	$p_{m}$ (kPa)	Δp (Pa)	${\dot{m}}_{CV}$ (kg/s)	Ma (–)	Kn (–)
Figure 13a	10	27.38 ± 1.5%	528.4 ± 0.6%	2.63 × 10^−5^ ± 1.6%	0.039 ± 0.15%	1.85 × 10^−4^ ± 1.5%
Figure 13b	25	28.83 ± 1.5%	538.8 ± 0.6%	2.79 × 10^−5^ ± 1.6%	0.039 ± 0.15%	1.75 × 10^−4^ ± 1.5%
Figure 13c	50	30.36 ± 1.5%	549.9 ± 0.6%	2.95 × 10^−5^ ± 1.6%	0.039 ± 0.15%	1.66 × 10^−4^ ± 1.5%

**Table 2:** Flow properties associated to the images of Figure 15. The reported percentage change indicates how much the properties vary inside the group of $N_{i}$ = 20 average images.

Image	Δt (µs)	$p_{m}$ (Pa)	Δp (Pa)	${\dot{m}}_{CV}$ (kg/s)	Ma (–)	Kn (–)
Figure 15a	20	6904 ± 1.2%	371 ± 0.3%	5.4 × 10^−6^ ± 1.5%	0.032 ± 0.27%	7.3 × 10^−4^ ± 1.2%
Figure 15b	30	6620 ± 1.2%	367 ± 0.31%	5.2 × 10^−6^ ± 1.5%	0.031 ± 0.29%	7.3 × 10^−4^ ± 1.2%
Figure 15c	40	6337 ± 1.2%	362.8 ± 0.31%	4.9 × 10^−6^ ± 1.5%	0.031 ± 0.3%	8 × 10^−4^ ± 1.2%
Figure 15d	50	6071 ± 1.2%	358.6 ± 0.31%	4.6 × 10^−6^ ± 1.5%	0.031 ± 0.31%	8.3 × 10^−4^ ± 1.2%

**Table 3:** Flow properties associated to the images of Figure 17. The reported percentage change indicates how much the properties vary inside the group of $N_{i}$ = 20 average images.

Image	Δt (µs)	$p_{m}$ (Pa)	Δp (Pa)	${\dot{m}}_{CV}$ (kg/s)	Ma (–)	Kn (–)
Figure 17a	20	1576 ± 3.6%	1789.2 ± 3.2%	1.7 × 10^−6^ ± 6.5%	0.07 ± 2.7%	0.008 ± 3.5%
Figure 17b	30	1580 ± 3.6%	1793.2 ± 3.2%	1.7 × 10^−6^ ± 6.2%	0.07 ± 2.5%	0.008 ± 3.5%
Figure 17c	40	1576.6 ± 3.6%	1789.2 ± 3.2%	1.7 × 10^−6^ ± 6.4%	0.07 ± 2.7%	0.008 ± 3.5%
Figure 17d	50	1578.7 ± 3.6%	1791.8 ± 3.2%	1.7 × 10^−6^ ± 6%	0.07 ± 2.2%	0.008 ± 3.5%

**Table 4:** Flow properties associated to the images of Figure 18. The reported percentage change indicates how much the properties vary inside the group of $N_{i}$ = 20 average images.

Image	Δt (µs)	$p_{m}$ (Pa)	Δp (Pa)	${\dot{m}}_{CV}$ (kg/s)	Ma (–)	Kn (–)
Figure 18a	20	920 ± 2.3%	1092.7 ± 2.2%	6.5 × 10^−7^ ± 4.4%	0.045 ± 2%	0.014 ± 2.3%
Figure 18b	30	920.3 ± 2.3%	1093.4 ± 2.3%	6.5 × 10^−7^ ± 4%	0.045 ± 1.7%	0.014 ± 2.3%
Figure 18c	40	919.1 ± 2.3%	1091.8 ± 2.2%	6.5 × 10^−7^ ± 3.9%	0.045 ± 1.6%	0.014 ± 2.3%
Figure 18d	50	919.1 ± 2.4%	1090.7 ± 2.2%	6.5 × 10^−7^ ± 4.4%	0.045 ± 2%	0.014 ± 2.3%

**Table 5:** Thickness $\Delta s_{x}$ of the displacement profile related to the data of argon–acetone flow at 6 kPa (Figure 16a) and of helium–acetone flows at 1.6 kPa (Figure 18a) and at 920 Pa (Figure 18b).

Δt (µs)	Argon–acetone at 6.5 kPa	Helium–acetone at 1.6 kPa	Helium–acetone at 920 Pa
20	206 µm	384.7 µm	149.3 µm
30	298.3 µm	528.5 µm	159.3 µm
40	334.96 µm	748.3 µm	271.9 µm
50	398.35 µm	1124.98 µm	380.8 µm

**Table 6:** Average velocities ${\bar{u}}_{CV}$ and ${\bar{u}}_{MTV}$ with estimated uncertainties, and relative deviation between ${\bar{u}}_{CV}$ and ${\bar{u}}_{MTV}$ calculated for the image data of Figure 13. The uncertainty range has a confidence level of 95%.

Δt (µs)	${\bar{u}}_{CV}$ (m/s)	${\bar{u}}_{MTV}$ (m/s)	$\left({\bar{u}}_{CV}-{\bar{u}}_{MTV}\right)/{\bar{u}}_{MTV}$ (%)
10	12.42 ± 0.74	10.47 ± 3	18.6
25	12.48 ± 0.74	11.21 ± 1.2	11.3
50	12.54 ± 0.76	11.93 ± 0.6	5.1

**Table 7:** Average velocities ${\bar{u}}_{CV}$ and ${\bar{u}}_{MTV}$ with estimated uncertainties, and relative deviation between ${\bar{u}}_{CV}$ and ${\bar{u}}_{MTV}$ calculated for the image data of Figure 15. The uncertainty range has a confidence level of 95%.

Δt (µs)	${\bar{u}}_{CV}$ (m/s)	${\bar{u}}_{MTV}$ (m/s)	$\left({\bar{u}}_{CV}-{\bar{u}}_{MTV}\right)/{\bar{u}}_{MTV}$ (%)
20	10.16 ± 0.6	9.08 ± 1.52	11.9
30	10.06 ± 0.6	9.83 ± 0.98	2.3
40	9.95 ± 0.6	9.92 ± 0.76	0.3
50	9.84 ± 0.6	9.9 ± 0.6	0.6

**Table 8:** Average velocities ${\bar{u}}_{CV}$ and ${\bar{u}}_{MTV}$ with estimated uncertainties, and relative deviation between ${\bar{u}}_{CV}$ and ${\bar{u}}_{MTV}$ calculated for the image data of Figure 17. The uncertainty range has a confidence level of 95%.

Δt (µs)	${\bar{u}}_{CV}$ (m/s)	${\bar{u}}_{MTV}$ (m/s)	$\left({\bar{u}}_{CV}-{\bar{u}}_{MTV}\right)/{\bar{u}}_{MTV}$ (%)
20	41.2 ± 2.48	44.59 ± 1.52	7.6
30	41.35 ± 2.48	44.47 ± 0.98	7
40	41.28 ± 2.48	43.78 ± 0.76	5.7
50	41.15 ± 2.46	43.09 ± 0.6	4.5

**Table 9:** Average velocities ${\bar{u}}_{CV}$ and ${\bar{u}}_{MTV}$ with estimated uncertainties, and relative deviation between ${\bar{u}}_{CV}$ and ${\bar{u}}_{MTV}$ calculated for the image data of Figure 18. The uncertainty range has a confidence level of 95%.

Δt (µs)	${\bar{u}}_{CV}$ (m/s)	${\bar{u}}_{MTV}$ (m/s)	$\left({\bar{u}}_{CV}-{\bar{u}}_{MTV}\right)/{\bar{u}}_{MTV}$ (%)
20	26.72 ± 1.6	31.06 ± 1.52	14
30	26.6 ± 1.6	31.24 ± 0.98	14.8
40	26.7 ± 1.6	29.81 ± 0.76	10.4
50	26.77 ± 1.6	28.89 ± 0.6	7.3
